# Prevalence and reclassification of *BRCA1* and *BRCA2* variants in a large, unselected Chinese Han breast cancer cohort

**DOI:** 10.1186/s13045-020-01010-0

**Published:** 2021-01-18

**Authors:** Yun Liu, Honglian Wang, Xin Wang, Jiaqi Liu, Junjian Li, Xiang Wang, Yun Zhang, Zhigang Bai, Qinghua Zhou, Ying Wu, Yi Shen, Xiaoling Weng, Fatao Liu, Jiancheng Guo, Lijun Di, Olivier Gires, Zhongtao Zhang, Yiding Chen, Hongxia Wang

**Affiliations:** 1 State Key Laboratory of Oncogenes and Related Genes, Department of Oncology, Shanghai General Hospital, Shanghai Jiao Tong University School of Medicine, 100 Haining Road, Shanghai, China; 2 State Key Laboratory of Oncogenes and Related Genes, Shanghai Cancer Institute, Renji Hospital, School of Medicine, Shanghai Jiao Tong University, Shanghai, China; 3 Department of Biliary-Pancreatic Surgery, Renji Hospital, School of Medicine, Shanghai Jiao Tong University, Shanghai, China; 4 AITA Biomedical Research Institute, Shanghai, China; 5 Department of Breast Surgical Oncology, National Cancer Center/National Clinical Research Center for Cancer/Cancer Hospital, Chinese Academy of Medical Sciences and Peking Union Medical College, Beijing, China; 6 Department of General Surgery, Beijing Friendship Hospital, Capital Medical University, 95 Yong’an Road, Beijing, China; 7 Beijing Key Laboratory of Cancer Invasion and Metastasis Research and National Clinical Research Center for Digestive Diseases, Beijing, China; 8 Department of Surgery, Luwan Branch of Ruijin Hospital, Shanghai Jiao Tong University School of Medicine, Shanghai, China; 9 Shanghai Key Laboratory of Biliary Tract Disease Research, Shanghai, China; 10 Center for Precision Medicine, Zhengzhou University School of Medicine, Zhengzhou, China; 11 Cancer Center, Faculty of Health Science, University of Macau, Macau, China; 12 Department of Otorhinolaryngology, Grosshadern Medical Center, Ludwig Maximilians University of Munich, Munich, Germany; 13 Department of Surgical Oncology, The Second Affiliated Hospital, Zhejiang University School of Medicine, 88 Jiefang Road, Hangzhou, Zhejiang China; 14 The Key Laboratory of Cancer Prevention and Intervention, China National Ministry of Education, Hangzhou, Zhejiang China

**Keywords:** Breast cancer, Cohort, *BRCA1/2*, VUS, Reclassification

## Abstract

Accurate interpretation of *BRCA1/2* variants is critical for risk assessment and precise treatment of breast cancer (BC). Hence, the establishment of an ethnicity-based *BRCA1/2* variant database of the Chinese population is of paramount importance. In this study, panel-based sequencing served to detect *BRCA1/*2 variants in a Chinese multicenter cohort of 21,216 BC patients and 6434 healthy controls. Overall, the percentage of subjects carrying pathogenic variants was 5.5% (1174/21,216) in BC patients and 1.1% (71/6434) in healthy controls. We identified 13 pathogenic variants as high-frequency variants that had a frequency of > 0.45‰ in BC patients (≥ 10 in 21,216 patients), none of which has been reported in Caucasians. Pathogenic *BRCA1/2* variants correlated with younger onset age, higher frequencies of bilateral and triple-negative BC (TNBC), invasive carcinomas, high histological grades, and family history of BC and other cancers. Furthermore, the percentage of the subjects carrying VUS was 9.8% (2071/21,216) in BC patients and 6.9% (446/6434) in healthy controls. Based on our cohort study, we unambiguously reclassified 7 out of the 858 VUS resulting in lower VUS ratio in patients (from 9.8 to 7.9%) as well as in healthy control (from 6.9 to 5.3%). We also re-analyzed the 100 variants in 13 exons (2–5 and 15–23) of the *BRCA1* genes using a functional assay (saturation genome editing; SGE). 55 of the 59 VUS had distinct status in the SGE study: 24 (43.6%) were pathogenic, and 31 (56.4%) were benign. Strong ethnicity-specific occurrences of pathogenic *BRCA1/2* variants were identified in the Chinese population. Hence, the findings provide rationale and sequencing information for the implementation of *BRCA1/2* variants tailored to the Chinese population into clinical risk assessment.

**To the Editor,**

Accurate interpretation of *BRCA1* and *BRCA2* variants is important for risk assessment and treatment of BC. Currently, available databases of *BRCA1/2* variants are mainly derived from the Caucasian population and may not be suitable for use in the Chinese population due to considerable ethnic differences. In a previous study, Sun et al*.* examined *BRCA1/2* variants in 8085 Chinese BC patients, however without the inclusion of healthy controls in the study [1]. During a period from 10-01-2015 to 12-15-2018, we collected 21,216 unselected Chinese BC patients and 6434 healthy controls in 19 medical centers in 11 Chinese provinces (Additional file 1: Fig. S1). Subjects and methods are shown in detail in the Additional file 2. Panel-based sequencing identified a total of 1958 *BRAC1/2* variants. Based on the ClinVar database (clinvar_20171002.vcf.gz) and ACMG guidelines, 532 (27.2%) variants are pathogenic, 858 (43.8%) are VUS, and the remaining 568 variants (29.0%) are benign (Additional file 3: Table S1).

Percentages of the subjects carrying pathogenic variants were 5.5% (1174/21,216) in BC patients and 1.1% (71/6434) in healthy controls (Additional file 3: Table S1). A complete list is presented in Additional file 4: Table S2. The following 13 pathogenic variants had a frequency of > 0.45‰ in BC patients (≥ 10 in 21,216 patients): p.Cys328fs, p.Asn704fs, p.Ser1862fs, and p.Ile1845fs in *BRCA1*; p.Ala938fs, p.Gln1037*, p.Ser1722fs, p.Tyr1894*, p.Leu1908fs, p.Glu2198fs, p.Ser2378*, p.Pro2802fs, and p.Thr3033fs in *BRCA2*. Among these 13 variants, 8 variants are reported for the first time as high-frequency variants, none has been reported as high-frequency variants in Caucasians, one (p.Cys328fs) has been reported at high frequency in Korean patients [2] (Fig. 1), and the remaining 4 variants (p.Ser1862fs, p.Ile1845fs, p.Gln1037*, p.Tyr1894*) have been reported at high-frequency in other Chinese studies [1, 3].

**Fig. 1:**
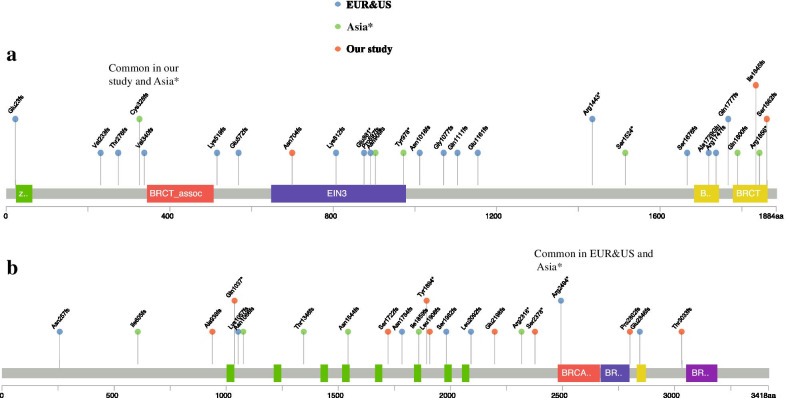
High-frequency *BRCA1/2* pathogenic variants distribution in Europe and USA, Asia*, and our study. Europe and USA: Include Ashkenazi Jew, Icelander, Norwegian, Finns, Swede, French, Dutch, Italian, French-Canadian, Hispanics (South California), Hispanics (Columbia), Afro-American, South African; Asia*: Include Iraqi/Iranian Jew, Singaporean, Filipino, Pakistani, Japanese, and Korean. **a** High-frequency *BRCA1* pathogenic variants distribution in Europe and USA, Asia*and China. Domains are Zinc/Ring finger (green); Serine cluster domain (blue); BRCT domain (red); BRCT (C terminus) (yellow). Variants in different region are indicated by color: blue: Europe and USA; green: Asia*; red: our study. One dot represents one variant; gray line length represents the number of groups with the variant. **b** High-frequency *BRCA2* pathogenic variants distribution in Europe and USA, Asia*and China. Domains are BRCA repeats (green); BRCA helica (red); OB binding domain (blue); tower (yellow) and OB3 binding domain (purple). Variants in different region are indicated by color: blue: Europe and USA; green: Asia*; red: our study. One dot represents one variant; gray line length represents the number of groups with the variant

In comparison with patients without *BRCA1/2* pathogenic variants (*n* = 16,472), both the patients carrying *BRCA1* (*n* = 404) and *BRCA2* pathogenic variants (*n* = 544) were younger, and more likely of having higher histological grade, having invasive carcinoma vs. ductal carcinoma, and having a family history of BC. *BRCA1* pathogenic variants were associated with TNBC and bilateral lesions, whereas *BRCA2* pathogenic variants were associated with Luminal B type (Additional file 5: Fig. S2 and Additional file 6: Table S3).

Percentages of the subjects carrying VUS were 9.8% (2071/21,216) in BC patients and 6.9% (446/6434) in healthy controls (Additional file 3: Table S1). 7 out of the 858 VUS had > 0.1% allele frequency in the entire cohort and no statistical difference between the patients and controls in our cohort, and thus were re-grouped into benign variants (Additional file 7: Table S4). The re-classification resulted in lower VUS ratio in patients (from 9.8 to 7.9%) and healthy controls (from 6.9 to 5.3%).

We next re-analyzed the 100 variants in 13 exons (2–5 and 15–23) of the *BRCA1* gene using a functional assay (saturation genome editing; SGE), as reported by Findlay et al*.* [4]. Under the ClinVar database and ACMG guidelines, 38 were pathogenic, 59 were VUS, and the remaining 3 were benign. 2 of the 38 pathogenic variants had distinct status in the Findlay study: one was VUS and another was benign. 55 of the 59 VUS had distinct status in the Findlay study: 24 (43.6%) were pathogenic, and 31 (56.4%) were benign (Additional file 8: Table S5). Notably, the 24 pathogenic variants under the functional assay were detected in BC patients only in our cohort. All 3 benign variants were also considered benign in the Findlay study .

In comparison with the 101 BC patients having VUS in the 13 *BRCA1* exons under the ClinVar database and ACMG guidelines, subjects re-grouped to pathogenic variants by SGE (24 variants, 38 pts) had higher rate of TNBC (50% vs 34.3%, *p* = 0.465), higher rate of early onset (36.8% vs. 26.7%, *p* = 0.516), and higher rate of having family history of BC (15.8% vs 8.9%, *p* = 0.465). In contrast, subjects re-grouped from VUS to benign (31 variants, 58 pts) had a lower rate of TNBC (24.3% vs 34.3%, *p* = 0.569), lower rate of early onset (20.7% vs 26.7%, *p* = 0.630), and lower rate of family history of BC (5.2% vs 8.9%, *p* = 0.626) (Table 1).

**Table 1:** Distribution proportion of 3 groups of *BRCA1* variants carriers clinical characteristics

Variables	Re-Pathogenic	VUS	P1-value	Re-Benign	P2-value
No. of subjects	38	101		58	
Age at entry	46.47	47.96		49.01	
Age at diagnosis	44.76	46.27		47.22	
Early onset breast cancer	36.84% (14/38)	26.73% (27/101)	0.516	20.69% (12/58)	0.630
Location of cancer (both sides/one side)	0 (0/23)	1.47% (1/68)	1	2.38% (1/42)	1
Luminal A breast cancer	0 (0/28)	8.57% (6/70)	0.290	13.51% (5/37)	0.699
Luminal B breast cancer	42.86% (12/28)	50% (35/70)	0.853	54.05% (20/37)	0.959
Triple negative breast cancer	50% (14/28)	34.29% (24/70)	0.465	24.32% (9/37)	0.569
HER2-positive breast cancer	7.14% (2/28)	7.14% (5/70)	1	8.11% (3/37)	1
Family history of breast cancer	15.79% (6/38)	8.91% (9/101)	0.465	5.17% (3/58)	0.626
Family history of other cancers	18.42% (7/38)	15.84% (16/101)	0.956	15.52% (9/58)	1

VUS: Detected in our study and located in 13 exons (2–5 and 15–23) of the *BRCA1* genes under the ClinVar database and ACMG guidelines

Re-Pathogenic: Above-mentioned VUS, re-grouped to pathogenic variants by SGE

Re-benign: Above-mentioned VUS, re-grouped to benign variants by SGE

Early onset breast cancer: Breast cancer was determined by an age ≤ 40 years at diagnosis

In summary, the current study demonstrated distinct *BRCA1/2* variant profiles in Chinese patients with BC, as well as healthy donors, and suggested testing based on hotspots in Caucasian patients/population is not appropriate. Hence, there is a need to develop a classification system that categorizes the known variants into pathogenic, VUS, and benign in the Chinese population. The biological impact of variants in the literature, allele frequency in the Chinese patients, and the general Chinese population should be incorporated into this classification system.

## Supplementary Information

**Additional file 1: Figure S1.** Schematic representation of the study and major results. Clinical samples from Chinese breast cancer patients (BCs; *n* = 21,216) and healthy controls (HCs; *n* = 6434) were subjected to an amplicon-based next-generation sequencing of the *BRCA1/2* genes. A total of *n* = 17,420 BCs and *n* = 5890 HCs were implemented in the clinical analysis in a 3-tier classification system to determine pathogenic variants (*n* = 1245) and variants of uncertain significance (VUS; *n* = 2517) of the *BRCA1*/2 genes. The repartition of the pathogenic variants with respect to frameshifts, stop-codon gains, splicing variants, missense mutations, and start-codon losses are depicted on the right. The 48 moderate-frequency pathogenic variants (detected in ≥ 5 BC patients) represented 39.8% of all pathogenic variants. Reclassification of VUS allowed to reduce the VUS ratio from 9.1 to 6.8%. The data demonstrate a high level of ethnicity-specific *BRCA1/2* germline mutations in the Chinese population compared to the Caucasian group.

**Additional file 2**. Subjects and methods.

**Additional file 3: Table S1.** Distribution of 3-tier-classified variants in BCs and HCs.

**Additional file 4: Table S2.** Carrier frequency of pathogenic variants in BCs and HCs.

**Additional file 5: Figure S2.** Comparison of clinical characteristics of BC patients with pathogenic *BRCA1/2* variants and BC patients with benign *BRCA1/2* variants/VUS. **a**. The distribution of age at diagnosis between pathogenic *BRCA1/2* variants carriers and benign *BRCA1/2* variants/VUS carriers. **b**. The distribution of BMI between pathogenic *BRCA1/2* variants carriers and benign *BRCA1/2* variants/VUS carriers. **c**. The distribution of histology between pathogenic *BRCA1/2* variants carriers and benign *BRCA1/2* variants/VUS carriers. **d**. The distribution of subtype between pathogenic *BRCA1/2* variants carriers and benign *BRCA1/2* variants/VUS carriers. **e**. The distribution of tumor size between pathogenic *BRCA1/2* variants carriers and benign *BRCA1/2* variants/VUS carriers. **f**. The distribution of histological grade between pathogenic *BRCA1/2* variants carriers and benign *BRCA1/2* variants/VUS carriers. **g**. The distribution of lymph modes status between pathogenic *BRCA1/2* variants carriers and benign *BRCA1/2* variants/VUS carriers. **h**. The distribution of location of cancer between pathogenic *BRCA1/2* variants carriers and benign *BRCA1/2* variants/VUS carriers. **i**. The distribution of family history of breast cancer between pathogenic *BRCA1/2* variants carriers and benign *BRCA1/2* variants/VUS carriers. **j**. The distribution of family history of other cancer between pathogenic *BRCA1/2* variants carriers and benign *BRCA1/2* variants/VUS carriers.

**Additional file 6: Table S3.** Clinical characteristics of BC patients with pathogenic *BRCA1/2* variants carriers and non-pathogenic variants carrier.

**Additional file 7: Table S4.** Variants detected in our study whose clinical significance were benign but conflicting interpretations of pathogenicity in ClinVar.

**Additional file 8: Table S5.** 55 VUS detected in our study with distinct status in the Findlay et al. study.

## Data Availability

All supporting data are included in the manuscript and supplemental files. Additional data are available upon reasonable request to the corresponding author.

## Abbreviations

BC: Breast cancer
VUS: Variants of uncertain significance
SGE: Saturation genome editing
TNBC: Triple negative breast cancer
SNV: Single-nucleotide variants
