# Supplementary figures and images for: Prevalence and reclassification of BRCA1 and BRCA2 variants in a large, unselected Chinese Han breast cancer cohort
Source: J Hematol Oncol. 2021 Jan 18;14:18. doi: 10.1186/s13045-020-01010-0 (PMC7814423; doi:10.1186/s13045-020-01010-0)

Suppl. Figure 1

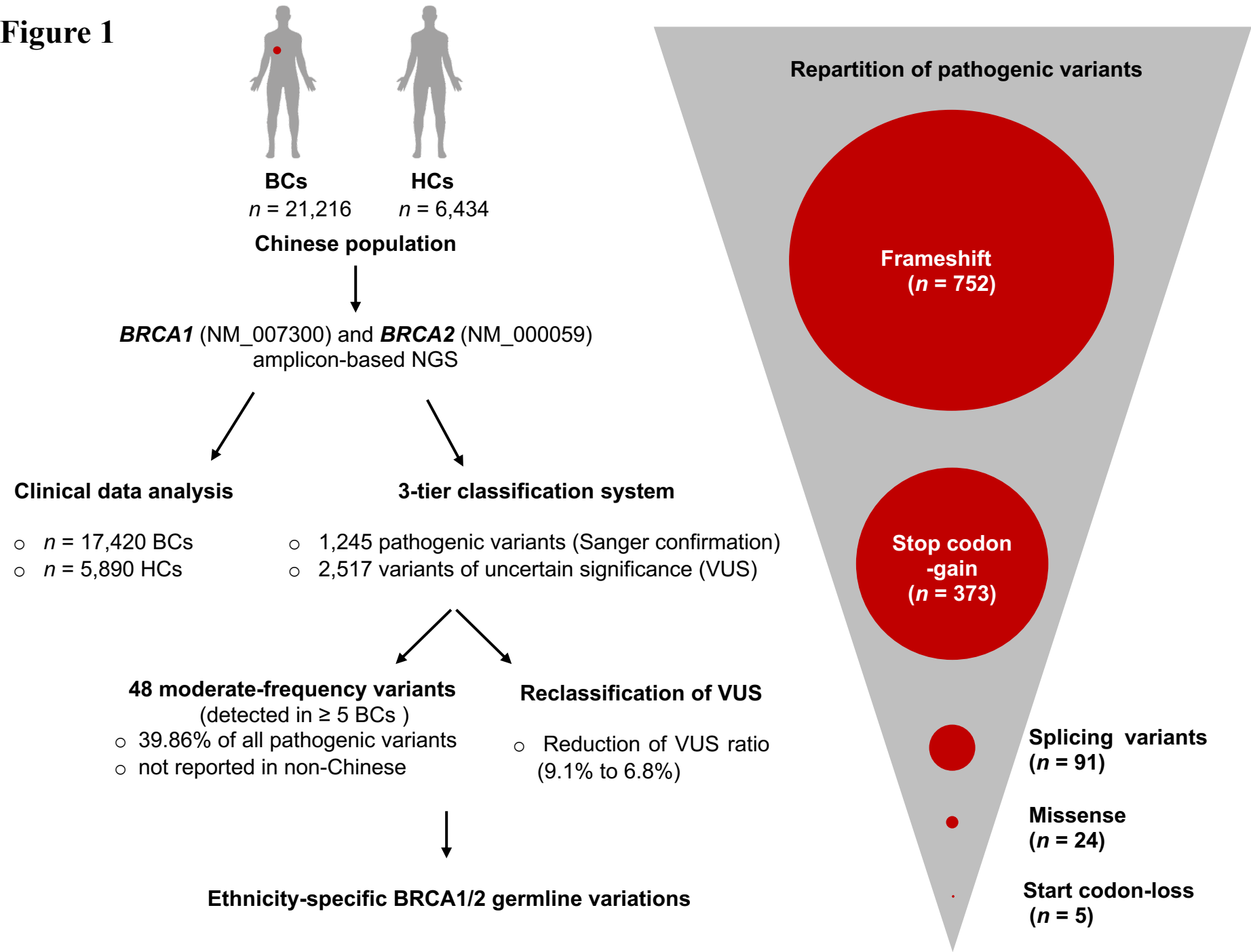

Supplement: Supplementary file 1 — Additional file 1: Figure S1. Schematic representation of the study and major results. Clinical samples from Chinese breast cancer patients (BCs; n = 21,216) and healthy controls (HCs; n = 6434) were subjected to an amplicon-based next-generation sequencing of the BRCA1/2 genes. A total of n = 17,420 BCs and n = 5890 HCs were implemented in the clinical analysis in a 3-tier classification system to determine pathogenic variants (n = 1245) and variants of uncertain significance (VUS; n = 2517) of the BRCA1/2 genes. The repartition of the pathogenic variants with respect to frameshifts, stop-codon gains, splicing variants, missense mutations, and start-codon losses are depicted on the right. The 48 moderate-frequency pathogenic variants (detected in ≥ 5 BC patients) represented 39.8% of all pathogenic variants. Reclassification of VUS allowed to reduce the VUS ratio from 9.1 to 6.8%. The data demonstrate a high level of ethnicity-specific BRCA1/2 germline mutations in the Chinese population compared to the Caucasian group. [file 13045_2020_1010_MOESM1_ESM.pdf]

Suppl. Figure 2

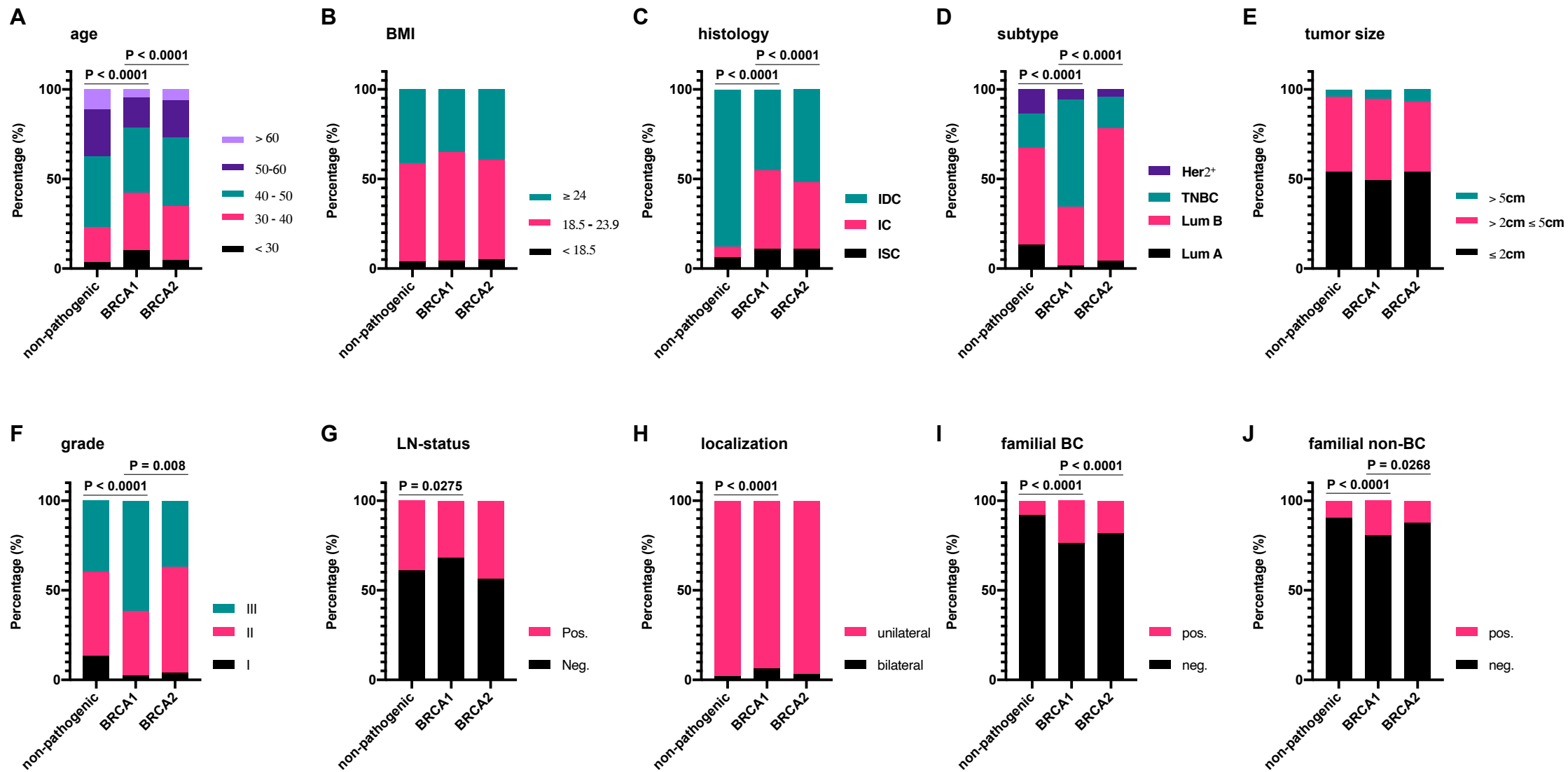

Supplement: Supplementary file 5 — Additional file 5: Figure S2. Comparison of clinical characteristics of BC patients with pathogenic BRCA1/2 variants and BC patients with benign BRCA1/2 variants/VUS. a. The distribution of age at diagnosis between pathogenic BRCA1/2 variants carriers and benign BRCA1/2 variants/VUS carriers. b. The distribution of BMI between pathogenic BRCA1/2 variants carriers and benign BRCA1/2 variants/VUS carriers. c. The distribution of histology between pathogenic BRCA1/2 variants carriers and benign BRCA1/2 variants/VUS carriers. d. The distribution of subtype between pathogenic BRCA1/2 variants carriers and benign BRCA1/2 variants/VUS carriers. e. The distribution of tumor size between pathogenic BRCA1/2 variants carriers and benign BRCA1/2 variants/VUS carriers. f. The distribution of histological grade between pathogenic BRCA1/2 variants carriers and benign BRCA1/2 variants/VUS carriers. g. The distribution of lymph modes status between pathogenic BRCA1/2 variants carriers and benign BRCA1/2 variants/VUS carriers. h. The distribution of location of cancer between pathogenic BRCA1/2 variants carriers and benign BRCA1/2 variants/VUS carriers. i. The distribution of family history of breast cancer between pathogenic BRCA1/2 variants carriers and benign BRCA1/2 variants/VUS carriers. j. The distribution of family history of other cancer between pathogenic BRCA1/2 variants carriers and benign BRCA1/2 variants/VUS carriers. [file 13045_2020_1010_MOESM5_ESM.pdf]
