# Supplementary material for: Prevalence and reclassification of BRCA1 and BRCA2 variants in a large, unselected Chinese Han breast cancer cohort
Source: J Hematol Oncol. 2021 Jan 18;14:18. doi: 10.1186/s13045-020-01010-0 (PMC7814423; doi:10.1186/s13045-020-01010-0)
Supplement: Supplementary file 2 — Additional file 2. Subjects and methods. [file 13045_2020_1010_MOESM2_ESM.docx]

**Subjects and methods**

***Subjects***

The study was approved by the Ethics Committee of all the hospitals involved and was performed according to the Declaration of Helsinki Principles. Written consent to notify blood samples for future research was obtained for each patient under the Institutional Review Board (IRB)-approved protocol.

Cases were unselected for age at diagnosis and family history of breast cancer (*n* = 21,216). Controls were unrelated, female donors without any reported oncological disease (*n* = 6,434). A family history of breast cancer was determined as individuals with one or more breast cancer patients among first-, second-, or third-degree relatives. Early-onset breast cancer was determined by an age ≤ 40 years at diagnosis (7). Estrogen receptor (ER), progesterone receptor (PR), and human epidermal growth factor receptor 2 (HER2) status were determined using BC tissue obtained from core-needle biopsies or surgery at the local hospital. For those in doubt were rechecked centrally. ER or PR immunostaining was considered positive at ≥ 1% nuclear staining (8). HER2-positivity was defined as a score of 3+ by immunohistochemical staining or HER2 gene amplification via fluorescence in situ hybridization (FISH) as described elsewhere (9). BCs were divided into luminal A, luminal B, triple-negative (TNBC), and HER2-positive (HER2^+^) subtypes (10-11). DNA samples were collected from subjects, and *BRCA1* and *BRCA2* genes (exons and exon-intron boundaries ± 20bp) were sequenced.

***Panel-based sequencing***

Target-specific primers for coding sequences of BRCA1 (NM_007300) and BRCA2 (NM_000059) were designed using Primer3 (http://bioinfo.ut.ee/primer3/). Universal sequences (CS1: ACACTGACGACATGGTTCTACA and CS2: TACGGTAGCAGAGACTTGGTCT) were appended at 5’-ends of each forward and reverse primer, respectively. Preamplification for tagged amplicon deep sequencing (Tam-Seq) was conducted in 6 mL PCR mixture containing 3 mL of KAPA 2G Robust HotStart ReadyMix (2X) (Kapa Biosystems, Boston, Massachusetts, United States), 1 mL of primer mix (500 nM), and 2 mL of DNA template (10 ng/ml). Sequencing barcode primers (Fluidigm Corporation, South San Francisco, California) consisted of PE1 and PE2 sequences for Illumina cluster generation, a 10-bp barcode, and CS1 and CS2 adaptors, used in pairs (PE1-CS1/PE2-BC-CS2; PE1-CS2/ PE2-BC-CS1). For the DNA library, PCR products were barcoded and analyzed using gel electrophoresis to ensure the expected insertion size. The library was quantified by Agilent BioAnalyzer and sequenced using the Illumina Xten platform with paired-end reads of 150 bp *per* the manufacturer’s instructions. Custom sequencing primers for CS1 and CS2 targeted the paired reads, and 10-base indexing (barcode) reads *per* the recommendations of Fluidigm.

***Data processing and varianst calling***

Sequencing reads were aligned to the hg19 reference genome using BWA1 (http://bio-bwa.sourceforge.net). Genome Analysis Toolkit (GATK) 2 (<https://software.broadinstitute.org/gatk/>) was used for base quality score recalibration, indel realignment, and variant calling based on the following criteria: (i) QD < 2.0; (ii) MQ < 40.0; (iii) MQRankSum < -12.5; and (iv) ReadPosRankSum < -8.0. Variant functions were predicted using SnpEff (http://snpeff.sourceforge.net), PolyPhen-2 (http://genetics.bwh.harvard.edu/pph2/), PROVEAN (http://provean.jcvi.org/index.php), and SIFT (https://omictools.com/sift-tool). Variant population frequency was annotated with the ExAC database (http://exac.broadinstitute.org), the 1,000 Genomes database (https://www.internationalgenome.org), and an internal database.

***Variant interpretation***

Only novel *BRCA1/2* variants or variants with < 1% population frequency in 1,000 Genomes or ExAC were collected. Clinical significance of each variant was annotated according to ClinVar (<https://www.ncbi.nlm.nih.gov/clinvar/>) (12), ACMG-AMP guidelines (7), and other supporting evidence from the public literature and curated databases. Variants were manually inspected with Integrative Genomics Viewer (http://www.igv.org/), to exclude false-positives. Classfication of variants was collapsed from a 5-tier to 3-tier classification system comprised of pathogenic and benign variants, and VUS. All pathogenic variants were validated by Sanger sequencing.

***Data analyses***

Categorical variables were compared between variant carriers and non-carriers using the chi-squared test or Fisher’s exact test, where appropriate. P < 0.05 indicates statistical significance.

**References**

Musolino A , Bella M A , Bortesi B , et al. BRCA mutations, molecular markers, and clinical variables in early-onset breast cancer: A population-based study[J]. Breast. 2007, 16(3):280-292.

Hammond MEH, Hayes DF, Dowsett M, et al. American Society of Clinical Oncology/College of American pathologists guideline recommendations for immunohistochemical testing of estrogen and progesterone receptors in breast cancer. Arch pathol Lab Med. 2010;134(7):907–22.

Wolff AC, Hammond ME, Schwartz JN, et al. American Society of Clinical Oncology/College of American Pathologists guideline recommendations for human epidermal growth factor receptor 2 testing in breast cancer. J Clin Oncol. 2007;25(1):118–45.

Perou CM, Sørlie T, Eisen MB, van de Rijn M, Jeffrey SS, Rees CA, et al. Molecular portraits of human breast tumours. Nature. 2000 Aug 17;406(6797):747-52.

Hu Z, Fan C, Oh DS, Marron JS, He X, Qaqish BF, et al. The molecular portraints of breast tumors are conserved across microarray platforms.BMC Genomics. 2006 Apr 27;7:96.

[Landrum MJ](https://www.ncbi.nlm.nih.gov/pubmed/?term=Landrum%20MJ%5bAuthor%5d&cauthor=true&cauthor_uid=24234437), [Lee JM](https://www.ncbi.nlm.nih.gov/pubmed/?term=Lee%20JM%5bAuthor%5d&cauthor=true&cauthor_uid=24234437), [Riley GR](https://www.ncbi.nlm.nih.gov/pubmed/?term=Riley%20GR%5bAuthor%5d&cauthor=true&cauthor_uid=24234437), [Jang W](https://www.ncbi.nlm.nih.gov/pubmed/?term=Jang%20W%5bAuthor%5d&cauthor=true&cauthor_uid=24234437), [Rubinstein WS](https://www.ncbi.nlm.nih.gov/pubmed/?term=Rubinstein%20WS%5bAuthor%5d&cauthor=true&cauthor_uid=24234437), [Church DM](https://www.ncbi.nlm.nih.gov/pubmed/?term=Church%20DM%5bAuthor%5d&cauthor=true&cauthor_uid=24234437), et al. ClinVar: public archive of relationships among sequence variation and human phenotype. Nucleic Acids Res. 2014; 42 (Database issue): p. D980-5.
