# Supplementary material for: Prevalence and reclassification of BRCA1 and BRCA2 variants in a large, unselected Chinese Han breast cancer cohort
Source: J Hematol Oncol. 2021 Jan 18;14:18. doi: 10.1186/s13045-020-01010-0 (PMC7814423; doi:10.1186/s13045-020-01010-0)
Supplement: Supplementary file 3 — Additional file 3: Table S1. Distribution of 3-tier-classified variants in BCs and HCs. [file 13045_2020_1010_MOESM3_ESM.docx]

**Supplementary Table 1 Distribution of 3-tier-classified variants in BCs and HCs**

| **Variant classification** | **Variants carriers of BCs (%)** | **Variants carriers of HCs (%)** | **Distinct variant number** |
| --- | --- | --- | --- |
| **Benign** | 84.70% (17,971/21,216) | 91.96% (5,917/6,434) | 568 |
| **VUS** | 9.76% (2,071/21,216) | 6.93% (446/6,434) | 858 |
| **Pathogenic** | 5.53% (1,174/21,216) | 1.10% (71/6,434) | 532 |

BCs: Breast cancer patients; HCs: Healthy controls;

Variant classification: Based on the Clinvar database and ACMG guidelines;
