# Supplementary material for: Prevalence and reclassification of BRCA1 and BRCA2 variants in a large, unselected Chinese Han breast cancer cohort
Source: J Hematol Oncol. 2021 Jan 18;14:18. doi: 10.1186/s13045-020-01010-0 (PMC7814423; doi:10.1186/s13045-020-01010-0)
Supplement: Supplementary file 4 — Additional file 4: Table S2. Carrier frequency of pathogenic variants in BCs and HCs. [file 13045_2020_1010_MOESM4_ESM.doc]

**Supplementary Table 2 Carrier frequency of pathogenic variants in BCs and HCs**

| **Gene** | **Chr:posi** | **ref>alt** | **Type** | **hgvs_c** | **hgvs_p** | **Number (HCs)** | **Number (BCs)** | **Carrier**  **frequency (HCs)** | **Carrier frequency (BCs)** |
| --- | --- | --- | --- | --- | --- | --- | --- | --- | --- |
| **BRCA1** | 17:41197753 | TAG>T | frameshift_variant | c.5595_5596delCT | p.Tyr1866fs | 0 | 1 | 0 | 4.71E-05 |
| **BRCA1** | 17:41197762 | ACACTGTCCAACACC>A | frameshift_variant | c.5574_5587delGGTGTTGGACAGTG | p.Trp1858fs | 0 | 1 | 0 | 4.71E-05 |
| **BRCA1** | 17:41197765 | CT>C | frameshift_variant | c.5584delA | p.Ser1862fs | 3 | 16 | 0.000466273 | 0.000754148 |
| **BRCA1** | 17:41197778 | ACT>A | frameshift_variant | c.5570_5571delAG | p.Glu1857fs | 0 | 2 | 0 | 9.43E-05 |
| **BRCA1** | 17:41197784 | G>A | stop_gained | c.5566C>T | p.Arg1856* | 0 | 5 | 0 | 0.000235671 |
| **BRCA1** | 17:41197801 | TC>T | frameshift_variant | c.5548delG | p.Glu1850fs | 0 | 1 | 0 | 4.71E-05 |
| **BRCA1** | 17:41197809 | CTGCCCAAT>C | frameshift_variant | c.5533_5540delATTGGGCA | p.Ile1845fs | 11 | 80 | 0.00170966 | 0.003770739 |
| **BRCA1** | 17:41197820 | C>T | splice_acceptor_variant | c.5531-1G>A |  | 0 | 3 | 0 | 0.000141403 |
| **BRCA1** | 17:41199659 | C>T | splice_donor_variant | c.5530+1G>A |  | 0 | 4 | 0 | 0.000188537 |
| **BRCA1** | 17:41199682 | C>T | stop_gained | c.5508G>A | p.Trp1836* | 0 | 1 | 0 | 4.71E-05 |
| **BRCA1** | 17:41199686 | GC>G | frameshift_variant | c.5503delG | p.Ala1835fs | 0 | 1 | 0 | 4.71E-05 |
| **BRCA1** | 17:41201135 | T>A | splice_donor_variant | c.5469+3A>T |  | 0 | 1 | 0 | 4.71E-05 |
| **BRCA1** | 17:41201137 | C>T | splice_donor_variant | c.5469+1G>A |  | 0 | 1 | 0 | 4.71E-05 |
| **BRCA1** | 17:41201148 | GTGAA>G | frameshift_variant | c.5455_5458delTTCA | p.Phe1819fs | 0 | 1 | 0 | 4.71E-05 |
| **BRCA1** | 17:41201157 | G>GA | frameshift_variant | c.5449dupT | p.Ser1817fs | 0 | 1 | 0 | 4.71E-05 |
| **BRCA1** | 17:41201157 | G>T | stop_gained | c.5450C>A | p.Ser1817* | 0 | 1 | 0 | 4.71E-05 |
| **BRCA1** | 17:41201183 | A>ACACAGCTG | frameshift_variant | c.5416_5423dupCAGCTGTG | p.Gly1809fs | 0 | 1 | 0 | 4.71E-05 |
| **BRCA1** | 17:41201191 | G>A | stop_gained | c.5416C>T | p.Gln1806* | 0 | 1 | 0 | 4.71E-05 |
| **BRCA1** | 17:41201209 | G>A | stop_gained | c.5398C>T | p.Gln1800* | 0 | 1 | 0 | 4.71E-05 |
| **BRCA1** | 17:41201212 | C>T | splice_acceptor_variant | c.5396-1G>A |  | 0 | 3 | 0 | 0.000141403 |
| **BRCA1** | 17:41201213 | T>C | splice_acceptor_variant | c.5396-2A>G |  | 0 | 5 | 0 | 0.000235671 |
| **BRCA1** | 17:41201213 | T>G | splice_acceptor_variant | c.5396-2A>C |  | 0 | 1 | 0 | 4.71E-05 |
| **BRCA1** | 17:41203078 | A>AC | splice_donor_variant | c.5395+1dupG |  | 0 | 1 | 0 | 4.71E-05 |
| **BRCA1** | 17:41203079 | C>T | splice_donor_variant | c.5395+1G>A |  | 0 | 6 | 0 | 0.000282805 |
| **BRCA1** | 17:41203135 | C>G | splice_acceptor_variant | c.5341-1G>C |  | 0 | 4 | 0 | 0.000188537 |
| **BRCA1** | 17:41203135 | C>T | splice_acceptor_variant | c.5341-1G>A |  | 1 | 0 | 0.000155424 | 0 |
| **BRCA1** | 17:41209068 | C>T | splice_donor_variant | c.5340+1G>A |  | 1 | 2 | 0.000155424 | 9.43E-05 |
| **BRCA1** | 17:41209079 | T>TG | frameshift_variant | c.5329dupC | p.Gln1777fs | 0 | 1 | 0 | 4.71E-05 |
| **BRCA1** | 17:41209095 | G>A | stop_gained | c.5314C>T | p.Arg1772* | 1 | 9 | 0.000155424 | 0.000424208 |
| **BRCA1** | 17:41209107 | G>A | stop_gained | c.5302C>T | p.Gln1768* | 0 | 3 | 0 | 0.000141403 |
| **BRCA1** | 17:41209133 | CCT>C | frameshift_variant | c.5274_5275delAG | p.Gly1759fs | 0 | 1 | 0 | 4.71E-05 |
| **BRCA1** | 17:41215349 | C>A | splice_donor_variant | c.5256+1G>T |  | 0 | 2 | 0 | 9.43E-05 |
| **BRCA1** | 17:41215370 | C>CT | frameshift_variant | c.5235dupA | p.Glu1746fs | 0 | 1 | 0 | 4.71E-05 |
| **BRCA1** | 17:41215382 | G>A | stop_gained | c.5224C>T | p.Gln1742* | 0 | 1 | 0 | 4.71E-05 |
| **BRCA1** | 17:41215386 | CA>C | frameshift_variant | c.5219delT | p.Val1740fs | 0 | 1 | 0 | 4.71E-05 |
| **BRCA1** | 17:41215387 | AC>A | frameshift_variant | c.5218delG | p.Val1740fs | 1 | 1 | 0.000155424 | 4.71E-05 |
| **BRCA1** | 17:41215389 | C>T | stop_gained | c.5217G>A | p.Trp1739* | 0 | 3 | 0 | 0.000141403 |
| **BRCA1** | 17:41215391 | CT>C | splice_acceptor_variant | c.5216-2delA |  | 0 | 1 | 0 | 4.71E-05 |
| **BRCA1** | 17:41215888 | T>TA | splice_donor_variant | c.5215+2dupT |  | 0 | 1 | 0 | 4.71E-05 |
| **BRCA1** | 17:41215947 | C>T | missense_variant | c.5159G>A | p.Arg1720Gln | 0 | 2 | 0 | 9.43E-05 |
| **BRCA1** | 17:41215948 | G>A | missense_variant | c.5158C>T | p.Arg1720Trp | 0 | 1 | 0 | 4.71E-05 |
| **BRCA1** | 17:41215957 | C>CA | frameshift_variant | c.5148dupT | p.Val1717fs | 0 | 1 | 0 | 4.71E-05 |
| **BRCA1** | 17:41219623 | A>G | splice_donor_variant | c.5137+2T>C |  | 0 | 2 | 0 | 9.43E-05 |
| **BRCA1** | 17:41219625 | C>T | missense_variant | c.5137G>A | p.Asp1713Asn | 1 | 4 | 0.000155424 | 0.000188537 |
| **BRCA1** | 17:41219627 | G>T | missense_variant | c.5135C>A | p.Thr1712Lys | 0 | 2 | 0 | 9.43E-05 |
| **BRCA1** | 17:41219634 | TA>T | frameshift_variant | c.5127delT | p.Met1710fs | 0 | 3 | 0 | 0.000141403 |
| **BRCA1** | 17:41219665 | ATTAG>A | frameshift_variant | c.5093_5096delCTAA | p.Thr1698fs | 0 | 1 | 0 | 4.71E-05 |
| **BRCA1** | 17:41219714 | T>C | splice_acceptor_variant | c.5050-2A>G |  | 0 | 3 | 0 | 0.000141403 |
| **BRCA1** | 17:41222944 | C>A | splice_donor_variant | c.5049+1G>T |  | 0 | 3 | 0 | 0.000141403 |
| **BRCA1** | 17:41223043 | CT>C | frameshift_variant | c.4950delA | p.Glu1651fs | 0 | 1 | 0 | 4.71E-05 |
| **BRCA1** | 17:41223097 | G>A | stop_gained | c.4897C>T | p.Gln1633* | 0 | 1 | 0 | 4.71E-05 |
| **BRCA1** | 17:41223130 | T>A | stop_gained | c.4864A>T | p.Lys1622* | 1 | 6 | 0.000155424 | 0.000282805 |
| **BRCA1** | 17:41223180 | GCT>G | frameshift_variant | c.4812_4813delAG | p.Arg1604fs | 1 | 1 | 0.000155424 | 4.71E-05 |
| **BRCA1** | 17:41223183 | C>CT | frameshift_variant | c.4810dupA | p.Arg1604fs | 0 | 1 | 0 | 4.71E-05 |
| **BRCA1** | 17:41223214 | CAGAGA>C | frameshift_variant | c.4775_4779delTCTCT | p.Phe1592fs | 0 | 1 | 0 | 4.71E-05 |
| **BRCA1** | 17:41223218 | GA>G | frameshift_variant | c.4775delT | p.Phe1592fs | 0 | 1 | 0 | 4.71E-05 |
| **BRCA1** | 17:41223220 | AGAGGC>A | frameshift_variant | c.4769_4773delGCCTC | p.Ser1590fs | 0 | 1 | 0 | 4.71E-05 |
| **BRCA1** | 17:41223242 | G>C | stop_gained | c.4752C>G | p.Tyr1584* | 0 | 1 | 0 | 4.71E-05 |
| **BRCA1** | 17:41226367 | G>T | stop_gained | c.4719C>A | p.Tyr1573* | 0 | 1 | 0 | 4.71E-05 |
| **BRCA1** | 17:41226411 | G>A | stop_gained | c.4675C>T | p.Gln1559* | 0 | 4 | 0 | 0.000188537 |
| **BRCA1** | 17:41226428 | AC>A | frameshift_variant | c.4657delG | p.Val1553fs | 0 | 1 | 0 | 4.71E-05 |
| **BRCA1** | 17:41226450 | G>A | stop_gained | c.4636C>T | p.Gln1546* | 0 | 8 | 0 | 0.000377074 |
| **BRCA1** | 17:41226539 | C>G | splice_acceptor_variant | c.4548-1G>C |  | 0 | 1 | 0 | 4.71E-05 |
| **BRCA1** | 17:41226540 | T>G | splice_acceptor_variant | c.4548-2A>C |  | 0 | 2 | 0 | 9.43E-05 |
| **BRCA1** | 17:41228504 | C>A | splice_donor_variant | c.4547+1G>T |  | 0 | 3 | 0 | 0.000141403 |
| **BRCA1** | 17:41228601 | T>TA | frameshift_variant | c.4450dupT | p.Tyr1484fs | 0 | 1 | 0 | 4.71E-05 |
| **BRCA1** | 17:41228617 | G>A | stop_gained | c.4435C>T | p.Gln1479* | 0 | 1 | 0 | 4.71E-05 |
| **BRCA1** | 17:41231353 | G>GTT | frameshift_variant | c.4420_4421insAA | p.Ser1474fs | 0 | 2 | 0 | 9.43E-05 |
| **BRCA1** | 17:41231377 | GA>G | frameshift_variant | c.4396delT | p.Ser1466fs | 0 | 2 | 0 | 9.43E-05 |
| **BRCA1** | 17:41234439 | G>GTTCT | frameshift_variant | c.4335_4338dupAGAA | p.Gln1447fs | 0 | 1 | 0 | 4.71E-05 |
| **BRCA1** | 17:41234451 | G>A | stop_gained | c.4327C>T | p.Arg1443* | 1 | 1 | 0.000155424 | 4.71E-05 |
| **BRCA1** | 17:41234488 | A>AG | frameshift_variant | c.4289dupC | p.Ser1431fs | 0 | 1 | 0 | 4.71E-05 |
| **BRCA1** | 17:41234520 | G>A | stop_gained | c.4258C>T | p.Gln1420* | 0 | 1 | 0 | 4.71E-05 |
| **BRCA1** | 17:41242960 | C>T | splice_donor_variant | c.4185+1G>A |  | 0 | 4 | 0 | 0.000188537 |
| **BRCA1** | 17:41242970 | TA>T | frameshift_variant | c.4175delT | p.Leu1392fs | 0 | 1 | 0 | 4.71E-05 |
| **BRCA1** | 17:41242982 | C>CT | frameshift_variant | c.4163dupA | p.Ser1389fs | 0 | 1 | 0 | 4.71E-05 |
| **BRCA1** | 17:41242983 | TG>T | frameshift_variant | c.4162delC | p.Gln1388fs | 0 | 2 | 0 | 9.43E-05 |
| **BRCA1** | 17:41243012 | GACGCTTGTTTCACTCTCAC>G | frameshift_variant | c.4115_4133delGTGAGAGTGAAACAAGCGT | p.Cys1372fs | 0 | 1 | 0 | 4.71E-05 |
| **BRCA1** | 17:41243016 | CT>C | frameshift_variant | c.4129delA | p.Ser1377fs | 0 | 1 | 0 | 4.71E-05 |
| **BRCA1** | 17:41243029 | C>A | stop_gained | c.4117G>T | p.Glu1373* | 0 | 1 | 0 | 4.71E-05 |
| **BRCA1** | 17:41243043 | GC>G | frameshift_variant | c.4102delG | p.Ala1368fs | 0 | 1 | 0 | 4.71E-05 |
| **BRCA1** | 17:41243051 | T>C | splice_acceptor_variant | c.4097-2A>G |  | 1 | 0 | 0.000155424 | 0 |
| **BRCA1** | 17:41243450 | A>G | splice_donor_variant | c.4096+2T>C |  | 0 | 1 | 0 | 4.71E-05 |
| **BRCA1** | 17:41243454 | A>C | stop_gained | c.4094T>G | p.Leu1365* | 0 | 1 | 0 | 4.71E-05 |
| **BRCA1** | 17:41243465 | CAT>C | frameshift_variant | c.4081_4082delAT | p.Met1361fs | 0 | 1 | 0 | 4.71E-05 |
| **BRCA1** | 17:41243479 | CTTGA>C | frameshift_variant | c.4065_4068delTCAA | p.Asn1355fs | 0 | 1 | 0 | 4.71E-05 |
| **BRCA1** | 17:41243533 | C>A | stop_gained | c.4015G>T | p.Glu1339* | 0 | 1 | 0 | 4.71E-05 |
| **BRCA1** | 17:41243534 | CT>C | frameshift_variant | c.4013delA | p.Lys1338fs | 0 | 1 | 0 | 4.71E-05 |
| **BRCA1** | 17:41243581 | G>A | stop_gained | c.3967C>T | p.Gln1323* | 0 | 1 | 0 | 4.71E-05 |
| **BRCA1** | 17:41243599 | AG>A | frameshift_variant | c.3948delC | p.Leu1317fs | 0 | 1 | 0 | 4.71E-05 |
| **BRCA1** | 17:41243611 | G>A | stop_gained | c.3937C>T | p.Gln1313* | 0 | 1 | 0 | 4.71E-05 |
| **BRCA1** | 17:41243638 | C>A | stop_gained | c.3910G>T | p.Glu1304* | 0 | 1 | 0 | 4.71E-05 |
| **BRCA1** | 17:41243653 | G>A | stop_gained | c.3895C>T | p.Gln1299* | 0 | 1 | 0 | 4.71E-05 |
| **BRCA1** | 17:41243688 | TC>T | frameshift_variant | c.3859delG | p.Glu1287fs | 0 | 1 | 0 | 4.71E-05 |
| **BRCA1** | 17:41243707 | G>A | stop_gained | c.3841C>T | p.Gln1281* | 0 | 3 | 0 | 0.000141403 |
| **BRCA1** | 17:41243776 | CCT>C | frameshift_variant | c.3770_3771delAG | p.Glu1257fs | 0 | 8 | 0 | 0.000377074 |
| **BRCA1** | 17:41243830 | G>A | stop_gained | c.3718C>T | p.Gln1240* | 0 | 1 | 0 | 4.71E-05 |
| **BRCA1** | 17:41243843 | GT>G | frameshift_variant | c.3704delA | p.Asn1235fs | 0 | 3 | 0 | 0.000141403 |
| **BRCA1** | 17:41243843 | GTTTAC>G | frameshift_variant | c.3700_3704delGTAAA | p.Val1234fs | 0 | 1 | 0 | 4.71E-05 |
| **BRCA1** | 17:41243859 | A>T | stop_gained | c.3689T>A | p.Leu1230* | 0 | 2 | 0 | 9.43E-05 |
| **BRCA1** | 17:41243887 | C>A | stop_gained | c.3661G>T | p.Glu1221* | 0 | 2 | 0 | 9.43E-05 |
| **BRCA1** | 17:41243891 | CT>C | frameshift_variant | c.3656delA | p.Glu1219fs | 0 | 1 | 0 | 4.71E-05 |
| **BRCA1** | 17:41243911 | C>A | stop_gained | c.3637G>T | p.Glu1213* | 1 | 2 | 0.000155424 | 9.43E-05 |
| **BRCA1** | 17:41243920 | C>CT | frameshift_variant | c.3627dupA | p.Glu1210fs | 0 | 2 | 0 | 9.43E-05 |
| **BRCA1** | 17:41243941 | G>A | stop_gained | c.3607C>T | p.Arg1203* | 0 | 4 | 0 | 0.000188537 |
| **BRCA1** | 17:41243950 | G>A | stop_gained | c.3598C>T | p.Gln1200* | 1 | 2 | 0.000155424 | 9.43E-05 |
| **BRCA1** | 17:41244044 | AT>A | frameshift_variant | c.3503delA | p.Asn1168fs | 0 | 1 | 0 | 4.71E-05 |
| **BRCA1** | 17:41244076 | C>A | stop_gained | c.3472G>T | p.Glu1158* | 0 | 4 | 0 | 0.000188537 |
| **BRCA1** | 17:41244097 | CA>C | frameshift_variant | c.3450delT | p.Asp1151fs | 0 | 1 | 0 | 4.71E-05 |
| **BRCA1** | 17:41244105 | TC>T | frameshift_variant | c.3442delG | p.Glu1148fs | 1 | 5 | 0.000155424 | 0.000235671 |
| **BRCA1** | 17:41244140 | AG>A | frameshift_variant | c.3407delC | p.Pro1136fs | 0 | 1 | 0 | 4.71E-05 |
| **BRCA1** | 17:41244184 | TATTAA>T | frameshift_variant | c.3359_3363delTTAAT | p.Val1120fs | 1 | 4 | 0.000155424 | 0.000188537 |
| **BRCA1** | 17:41244196 | G>A | stop_gained | c.3352C>T | p.Gln1118* | 0 | 1 | 0 | 4.71E-05 |
| **BRCA1** | 17:41244208 | C>A | stop_gained | c.3340G>T | p.Glu1114* | 0 | 1 | 0 | 4.71E-05 |
| **BRCA1** | 17:41244214 | CT>C | frameshift_variant | c.3333delA | p.Glu1112fs | 0 | 1 | 0 | 4.71E-05 |
| **BRCA1** | 17:41244218 | C>CT | frameshift_variant | c.3329dupA | p.Gln1111fs | 0 | 2 | 0 | 9.43E-05 |
| **BRCA1** | 17:41244218 | CT>C | frameshift_variant | c.3329delA | p.Lys1110fs | 0 | 1 | 0 | 4.71E-05 |
| **BRCA1** | 17:41244251 | AG>A | frameshift_variant | c.3296delC | p.Pro1099fs | 0 | 1 | 0 | 4.71E-05 |
| **BRCA1** | 17:41244253 | GA>G | frameshift_variant | c.3294delT | p.Pro1099fs | 0 | 4 | 0 | 0.000188537 |
| **BRCA1** | 17:41244258 | CTT>C | frameshift_variant | c.3288_3289delAA | p.Leu1098fs | 0 | 1 | 0 | 4.71E-05 |
| **BRCA1** | 17:41244262 | GT>G | frameshift_variant | c.3285delA | p.Lys1095fs | 0 | 2 | 0 | 9.43E-05 |
| **BRCA1** | 17:41244264 | TTA>T | frameshift_variant | c.3282_3283delTA | p.Tyr1094fs | 1 | 0 | 0.000155424 | 0 |
| **BRCA1** | 17:41244318 | CCT>C | frameshift_variant | c.3228_3229delAG | p.Gly1077fs | 0 | 4 | 0 | 0.000188537 |
| **BRCA1** | 17:41244366 | AT>A | frameshift_variant | c.3181delA | p.Ile1061fs | 0 | 1 | 0 | 4.71E-05 |
| **BRCA1** | 17:41244602 | TG>T | frameshift_variant | c.2945delC | p.Pro982fs | 0 | 1 | 0 | 4.71E-05 |
| **BRCA1** | 17:41244634 | CAT>C | frameshift_variant | c.2912_2913delAT | p.His971fs | 0 | 1 | 0 | 4.71E-05 |
| **BRCA1** | 17:41244677 | CTGAGA>C | frameshift_variant | c.2866_2870delTCTCA | p.Ser956fs | 0 | 2 | 0 | 9.43E-05 |
| **BRCA1** | 17:41244738 | TTATC>T | frameshift_variant | c.2806_2809delGATA | p.Asp936fs | 0 | 1 | 0 | 4.71E-05 |
| **BRCA1** | 17:41244745 | TC>T | frameshift_variant | c.2802delG | p.Asp936fs | 0 | 1 | 0 | 4.71E-05 |
| **BRCA1** | 17:41244751 | CA>C | frameshift_variant | c.2796delT | p.Gly933fs | 0 | 2 | 0 | 9.43E-05 |
| **BRCA1** | 17:41244752 | AACCACAGG>A | frameshift_variant | c.2788_2795delCCTGTGGT | p.Pro930fs | 0 | 1 | 0 | 4.71E-05 |
| **BRCA1** | 17:41244844 | CAA>C | frameshift_variant | c.2702_2703delTT | p.Phe901fs | 0 | 1 | 0 | 4.71E-05 |
| **BRCA1** | 17:41244861 | CTT>C | frameshift_variant | c.2685_2686delAA | p.Pro897fs | 0 | 2 | 0 | 9.43E-05 |
| **BRCA1** | 17:41244865 | G>A | stop_gained | c.2683C>T | p.Gln895* | 0 | 1 | 0 | 4.71E-05 |
| **BRCA1** | 17:41244907 | C>A | stop_gained | c.2641G>T | p.Glu881* | 0 | 2 | 0 | 9.43E-05 |
| **BRCA1** | 17:41244945 | G>C | stop_gained | c.2603C>G | p.Ser868* | 0 | 1 | 0 | 4.71E-05 |
| **BRCA1** | 17:41244976 | G>A | stop_gained | c.2572C>T | p.Gln858* | 0 | 5 | 0 | 0.000235671 |
| **BRCA1** | 17:41245024 | C>A | stop_gained | c.2524G>T | p.Glu842* | 0 | 1 | 0 | 4.71E-05 |
| **BRCA1** | 17:41245056 | T>TA | frameshift_variant | c.2491dupT | p.Tyr831fs | 0 | 1 | 0 | 4.71E-05 |
| **BRCA1** | 17:41245060 | T>TA | frameshift_variant | c.2487dupT | p.Lys830fs | 0 | 1 | 0 | 4.71E-05 |
| **BRCA1** | 17:41245102 | G>GA | frameshift_variant | c.2445dupT | p.His816fs | 0 | 1 | 0 | 4.71E-05 |
| **BRCA1** | 17:41245114 | TG>T | frameshift_variant | c.2433delC | p.Lys812fs | 0 | 1 | 0 | 4.71E-05 |
| **BRCA1** | 17:41245118 | GT>G | frameshift_variant | c.2429delA | p.Asn810fs | 0 | 1 | 0 | 4.71E-05 |
| **BRCA1** | 17:41245210 | G>A | stop_gained | c.2338C>T | p.Gln780* | 2 | 1 | 0.000310849 | 4.71E-05 |
| **BRCA1** | 17:41245245 | CT>C | frameshift_variant | c.2302delA | p.Ser768fs | 0 | 5 | 0 | 0.000235671 |
| **BRCA1** | 17:41245253 | C>CT | frameshift_variant | c.2294dupA | p.Ser766fs | 0 | 1 | 0 | 4.71E-05 |
| **BRCA1** | 17:41245278 | AC>A | frameshift_variant | c.2269delG | p.Val757fs | 0 | 2 | 0 | 9.43E-05 |
| **BRCA1** | 17:41245346 | TTTCTC>T | frameshift_variant | c.2197_2201delGAGAA | p.Glu733fs | 0 | 1 | 0 | 4.71E-05 |
| **BRCA1** | 17:41245360 | C>A | stop_gained | c.2188G>T | p.Glu730* | 0 | 1 | 0 | 4.71E-05 |
| **BRCA1** | 17:41245366 | T>A | stop_gained | c.2182A>T | p.Arg728* | 0 | 1 | 0 | 4.71E-05 |
| **BRCA1** | 17:41245390 | C>CT | frameshift_variant | c.2157dupA | p.Glu720fs | 0 | 2 | 0 | 9.43E-05 |
| **BRCA1** | 17:41245390 | C>A | stop_gained | c.2158G>T | p.Glu720* | 0 | 1 | 0 | 4.71E-05 |
| **BRCA1** | 17:41245436 | ATT>A | frameshift_variant | c.2110_2111delAA | p.Asn704fs | 1 | 12 | 0.000155424 | 0.000565611 |
| **BRCA1** | 17:41245445 | CTT>C | frameshift_variant | c.2101_2102delAA | p.Lys701fs | 0 | 1 | 0 | 4.71E-05 |
| **BRCA1** | 17:41245579 | G>A | stop_gained | c.1969C>T | p.Gln657* | 0 | 1 | 0 | 4.71E-05 |
| **BRCA1** | 17:41245586 | C>CT | frameshift_variant | c.1961dupA | p.Tyr655fs | 1 | 3 | 0.000155424 | 0.000141403 |
| **BRCA1** | 17:41245586 | CT>C | frameshift_variant | c.1961delA | p.Lys654fs | 0 | 4 | 0 | 0.000188537 |
| **BRCA1** | 17:41245603 | C>A | stop_gained | c.1945G>T | p.Glu649* | 0 | 1 | 0 | 4.71E-05 |
| **BRCA1** | 17:41245847 | AT>A | frameshift_variant | c.1700delA | p.Asn567fs | 0 | 2 | 0 | 9.43E-05 |
| **BRCA1** | 17:41245878 | G>GT | frameshift_variant | c.1669dupA | p.Thr557fs | 0 | 1 | 0 | 4.71E-05 |
| **BRCA1** | 17:41245888 | C>A | stop_gained | c.1660G>T | p.Glu554* | 0 | 5 | 0 | 0.000235671 |
| **BRCA1** | 17:41245932 | G>GT | frameshift_variant | c.1615dupA | p.Thr539fs | 0 | 1 | 0 | 4.71E-05 |
| **BRCA1** | 17:41245939 | TA>T | frameshift_variant | c.1608delT | p.Asn537fs | 0 | 1 | 0 | 4.71E-05 |
| **BRCA1** | 17:41245948 | G>A | stop_gained | c.1600C>T | p.Gln534* | 0 | 1 | 0 | 4.71E-05 |
| **BRCA1** | 17:41245977 | GC>G | frameshift_variant | c.1570delG | p.Ala524fs | 0 | 1 | 0 | 4.71E-05 |
| **BRCA1** | 17:41246039 | CTTTAA>C | frameshift_variant | c.1504_1508delTTAAA | p.Leu502fs | 0 | 1 | 0 | 4.71E-05 |
| **BRCA1** | 17:41246040 | TTTAA>T | frameshift_variant | c.1504_1507delTTAA | p.Leu502fs | 0 | 1 | 0 | 4.71E-05 |
| **BRCA1** | 17:41246066 | TTGTA>T | frameshift_variant | c.1478_1481delTACA | p.Ile493fs | 0 | 1 | 0 | 4.71E-05 |
| **BRCA1** | 17:41246068 | G>A | stop_gained | c.1480C>T | p.Gln494* | 0 | 1 | 0 | 4.71E-05 |
| **BRCA1** | 17:41246077 | G>A | stop_gained | c.1471C>T | p.Gln491* | 0 | 5 | 0 | 0.000235671 |
| **BRCA1** | 17:41246083 | C>A | stop_gained | c.1465G>T | p.Glu489* | 2 | 3 | 0.000310849 | 0.000141403 |
| **BRCA1** | 17:41246127 | A>C | stop_gained | c.1421T>G | p.Leu474* | 0 | 1 | 0 | 4.71E-05 |
| **BRCA1** | 17:41246183 | ATTACTCTCTACTG>A | frameshift_variant | c.1352_1364delCAGTAGAGAGTAA | p.Ser451fs | 0 | 1 | 0 | 4.71E-05 |
| **BRCA1** | 17:41246206 | GAA>G | frameshift_variant | c.1340_1341delTT | p.Val447fs | 0 | 2 | 0 | 9.43E-05 |
| **BRCA1** | 17:41246208 | ACT>A | frameshift_variant | c.1338_1339delAG | p.Arg446fs | 0 | 1 | 0 | 4.71E-05 |
| **BRCA1** | 17:41246248 | T>TG | frameshift_variant | c.1299dupC | p.Ser434fs | 0 | 2 | 0 | 9.43E-05 |
| **BRCA1** | 17:41246296 | C>A | stop_gained | c.1252G>T | p.Glu418* | 0 | 1 | 0 | 4.71E-05 |
| **BRCA1** | 17:41246334 | G>C | stop_gained | c.1214C>G | p.Ser405* | 0 | 1 | 0 | 4.71E-05 |
| **BRCA1** | 17:41246415 | CT>C | frameshift_variant | c.1132delA | p.Ser378fs | 0 | 1 | 0 | 4.71E-05 |
| **BRCA1** | 17:41246433 | C>T | stop_gained | c.1115G>A | p.Trp372* | 0 | 1 | 0 | 4.71E-05 |
| **BRCA1** | 17:41246442 | TC>T | frameshift_variant | c.1105delG | p.Asp369fs | 0 | 1 | 0 | 4.71E-05 |
| **BRCA1** | 17:41246490 | C>T | stop_gained | c.1058G>A | p.Trp353* | 0 | 5 | 0 | 0.000235671 |
| **BRCA1** | 17:41246531 | C>CT | frameshift_variant | c.1016dupA | p.Val340fs | 0 | 1 | 0 | 4.71E-05 |
| **BRCA1** | 17:41246531 | CT>C | frameshift_variant | c.1016delA | p.Lys339fs | 0 | 3 | 0 | 0.000141403 |
| **BRCA1** | 17:41246560 | C>CAT | frameshift_variant | c.986_987dupAT | p.Asp330fs | 0 | 1 | 0 | 4.71E-05 |
| **BRCA1** | 17:41246565 | CAT>C | frameshift_variant | c.981_982delAT | p.Cys328fs | 0 | 14 | 0 | 0.000659879 |
| **BRCA1** | 17:41246585 | C>T | stop_gained | c.963G>A | p.Trp321* | 0 | 1 | 0 | 4.71E-05 |
| **BRCA1** | 17:41246586 | C>T | stop_gained | c.962G>A | p.Trp321* | 0 | 1 | 0 | 4.71E-05 |
| **BRCA1** | 17:41246594 | A>AAC | frameshift_variant | c.953_954insGT | p.His318fs | 0 | 1 | 0 | 4.71E-05 |
| **BRCA1** | 17:41246620 | G>A | stop_gained | c.928C>T | p.Gln310* | 0 | 1 | 0 | 4.71E-05 |
| **BRCA1** | 17:41246653 | CA>C | frameshift_variant | c.894delT | p.Asn298fs | 0 | 2 | 0 | 9.43E-05 |
| **BRCA1** | 17:41246678 | TA>T | frameshift_variant | c.869delT | p.Leu290fs | 0 | 1 | 0 | 4.71E-05 |
| **BRCA1** | 17:41246793 | CG>C | frameshift_variant | c.754delC | p.Arg252fs | 0 | 1 | 0 | 4.71E-05 |
| **BRCA1** | 17:41247864 | CT>C | frameshift_variant | c.668delA | p.Lys223fs | 1 | 0 | 0.000155424 | 0 |
| **BRCA1** | 17:41251811 | C>CGTCT | frameshift_variant | c.524_527dupAGAC | p.Ser177fs | 0 | 1 | 0 | 4.71E-05 |
| **BRCA1** | 17:41251819 | G>A | stop_gained | c.520C>T | p.Gln174* | 0 | 2 | 0 | 9.43E-05 |
| **BRCA1** | 17:41251834 | G>A | stop_gained | c.505C>T | p.Gln169* | 0 | 4 | 0 | 0.000188537 |
| **BRCA1** | 17:41256157 | TTC>T | frameshift_variant | c.421_422delGA | p.Glu141fs | 0 | 1 | 0 | 4.71E-05 |
| **BRCA1** | 17:41256192 | AG>A | frameshift_variant | c.387delC | p.Tyr130fs | 0 | 1 | 0 | 4.71E-05 |
| **BRCA1** | 17:41256212 | G>GA | frameshift_variant | c.367dupT | p.Ser123fs | 0 | 1 | 0 | 4.71E-05 |
| **BRCA1** | 17:41256218 | T>TC | frameshift_variant | c.361dupG | p.Glu121fs | 0 | 1 | 0 | 4.71E-05 |
| **BRCA1** | 17:41256244 | AT>A | frameshift_variant | c.335delA | p.Asn112fs | 0 | 3 | 0 | 0.000141403 |
| **BRCA1** | 17:41256250 | CTT>C | frameshift_variant | c.328_329delAA | p.Lys110fs | 0 | 1 | 0 | 4.71E-05 |
| **BRCA1** | 17:41256899 | TCAAG>T | frameshift_variant | c.283_286delCTTG | p.Leu95fs | 0 | 2 | 0 | 9.43E-05 |
| **BRCA1** | 17:41256906 | G>A | stop_gained | c.280C>T | p.Gln94* | 0 | 1 | 0 | 4.71E-05 |
| **BRCA1** | 17:41256933 | C>A | stop_gained | c.253G>T | p.Glu85* | 0 | 1 | 0 | 4.71E-05 |
| **BRCA1** | 17:41256966 | G>A | stop_gained | c.220C>T | p.Gln74* | 0 | 1 | 0 | 4.71E-05 |
| **BRCA1** | 17:41258472 | C>A | splice_donor_variant | c.212+1G>T |  | 0 | 2 | 0 | 9.43E-05 |
| **BRCA1** | 17:41258473 | C>T | missense_variant | c.212G>A | p.Arg71Lys | 0 | 6 | 0 | 0.000282805 |
| **BRCA1** | 17:41258496 | TA>T | frameshift_variant | c.188delT | p.Leu63fs | 0 | 1 | 0 | 4.71E-05 |
| **BRCA1** | 17:41258507 | G>A | stop_gained | c.178C>T | p.Gln60* | 0 | 2 | 0 | 9.43E-05 |
| **BRCA1** | 17:41258526 | GT>G | frameshift_variant | c.158delA | p.Asn53fs | 0 | 5 | 0 | 0.000235671 |
| **BRCA1** | 17:41258544 | G>T | stop_gained | c.141C>A | p.Cys47* | 0 | 1 | 0 | 4.71E-05 |
| **BRCA1** | 17:41258545 | C>CAA | frameshift_variant | c.138_139dupTT | p.Cys47fs | 0 | 1 | 0 | 4.71E-05 |
| **BRCA1** | 17:41258545 | C>A | missense_variant | c.140G>T | p.Cys47Phe | 0 | 1 | 0 | 4.71E-05 |
| **BRCA1** | 17:41258551 | CTATAAAT>C | splice_acceptor_variant | c.135-8_135-2delATTTATA | | 0 | 1 |  | 4.71E-05 |
| **BRCA1** | 17:41258552 | T>C | splice_acceptor_variant | c.135-2A>G |  | 0 | 3 | 0 | 0.000141403 |
| **BRCA1** | 17:41267761 | C>T | missense_variant | c.116G>A | p.Cys39Tyr | 0 | 1 | 0 | 4.71E-05 |
| **BRCA1** | 17:41267792 | CCA>C | frameshift_variant | c.83_84delTG | p.Leu28fs | 0 | 1 | 0 | 4.71E-05 |
| **BRCA1** | 17:41267798 | T>C | splice_acceptor_variant | c.81-2A>G |  | 0 | 1 | 0 | 4.71E-05 |
| **BRCA1** | 17:41276047 | C>CT | frameshift_variant | c.66dupA | p.Glu23fs | 0 | 4 | 0 | 0.000188537 |
| **BRCA1** | 17:41276080 | G>A | stop_gained | c.34C>T | p.Gln12* | 0 | 1 | 0 | 4.71E-05 |
| **BRCA1** | 17:41276111 | C>A | start_lost | c.3G>T | p.Met1? | 0 | 4 | 0 | 0.000188537 |
| **BRCA2** | 13:32890600 | G>A | start_lost | c.3G>A | p.Met1? | 0 | 1 | 0 | 4.71E-05 |
| **BRCA2** | 13:32890666 | T>C | splice_donor_variant | c.67+2T>C |  | 1 | 2 | 0.000155424 | 9.43E-05 |
| **BRCA2** | 13:32893239 | G>A | stop_gained | c.93G>A | p.Trp31* | 1 | 0 | 0.000155424 | 0 |
| **BRCA2** | 13:32893246 | G>T | stop_gained | c.100G>T | p.Glu34* | 0 | 1 | 0 | 4.71E-05 |
| **BRCA2** | 13:32893256 | C>G | stop_gained | c.110C>G | p.Ser37* | 0 | 1 | 0 | 4.71E-05 |
| **BRCA2** | 13:32893269 | CT>C | frameshift_variant | c.124delT | p.Tyr42fs | 0 | 1 | 0 | 4.71E-05 |
| **BRCA2** | 13:32893303 | A>T | stop_gained | c.157A>T | p.Lys53* | 0 | 1 | 0 | 4.71E-05 |
| **BRCA2** | 13:32893310 | AC>A | frameshift_variant | c.165delC | p.Asn55fs | 0 | 1 | 0 | 4.71E-05 |
| **BRCA2** | 13:32893315 | T>TA | frameshift_variant | c.170dupA | p.Tyr57fs | 0 | 1 | 0 | 4.71E-05 |
| **BRCA2** | 13:32893320 | AC>A | frameshift_variant | c.176delC | p.Pro59fs | 0 | 2 | 0 | 9.43E-05 |
| **BRCA2** | 13:32893342 | C>T | stop_gained | c.196C>T | p.Gln66* | 0 | 3 | 0 | 0.000141403 |
| **BRCA2** | 13:32893384 | ATATTC>A | frameshift_variant | c.239_243delTATTC | p.Ile80fs | 0 | 1 | 0 | 4.71E-05 |
| **BRCA2** | 13:32893388 | TC>T | frameshift_variant | c.243delC | p.Phe81fs | 0 | 1 | 0 | 4.71E-05 |
| **BRCA2** | 13:32893396 | C>T | stop_gained | c.250C>T | p.Gln84* | 0 | 1 | 0 | 4.71E-05 |
| **BRCA2** | 13:32893411 | C>CCGCT | frameshift_variant | c.266_269dupCGCT | p.Tyr91fs | 0 | 2 | 0 | 9.43E-05 |
| **BRCA2** | 13:32900244 | T>TG | frameshift_variant | c.433dupG | p.Val145fs | 0 | 2 | 0 | 9.43E-05 |
| **BRCA2** | 13:32900251 | C>T | stop_gained | c.439C>T | p.Gln147* | 0 | 2 | 0 | 9.43E-05 |
| **BRCA2** | 13:32900275 | AGAGAT>A | frameshift_variant | c.464_468delGAGAT | p.Arg155fs | 0 | 1 | 0 | 4.71E-05 |
| **BRCA2** | 13:32900280 | TAAGTC>T | frameshift_variant | c.470_474delAGTCA | p.Lys157fs | 1 | 9 | 0.000155424 | 0.000424208 |
| **BRCA2** | 13:32900420 | G>A | splice_donor_variant | c.516+1G>A |  | 0 | 1 | 0 | 4.71E-05 |
| **BRCA2** | 13:32900642 | C>T | stop_gained | c.523C>T | p.Gln175* | 0 | 1 | 0 | 4.71E-05 |
| **BRCA2** | 13:32900703 | C>G | stop_gained | c.584C>G | p.Ser195* | 0 | 1 | 0 | 4.71E-05 |
| **BRCA2** | 13:32900726 | AC>A | frameshift_variant | c.610delC | p.Ser205fs | 0 | 1 | 0 | 4.71E-05 |
| **BRCA2** | 13:32900731 | TAG>T | frameshift_variant | c.613_614delAG | p.Ser205fs | 1 | 1 | 0.000155424 | 4.71E-05 |
| **BRCA2** | 13:32900751 | G>A | splice_donor_variant | c.631+1G>A |  | 0 | 5 | 0 | 0.000235671 |
| **BRCA2** | 13:32903575 | TACAG>T | splice_acceptor_variant | c.632-4_632-1delACAG |  | 0 | 1 | 0 | 4.71E-05 |
| **BRCA2** | 13:32903579 | G>T | splice_acceptor_variant | c.632-1G>T |  | 0 | 2 | 0 | 9.43E-05 |
| **BRCA2** | 13:32903604 | CTG>C | frameshift_variant | c.658_659delGT | p.Val220fs | 0 | 1 | 0 | 4.71E-05 |
| **BRCA2** | 13:32905054 | A>G | splice_acceptor_variant | c.682-2A>G |  | 0 | 1 | 0 | 4.71E-05 |
| **BRCA2** | 13:32905094 | GA>G | frameshift_variant | c.722delA | p.Lys241fs | 0 | 3 | 0 | 0.000141403 |
| **BRCA2** | 13:32905098 | A>T | stop_gained | c.724A>T | p.Lys242* | 0 | 1 | 0 | 4.71E-05 |
| **BRCA2** | 13:32905137 | AAC>A | frameshift_variant | c.767_768delCA | p.Thr256fs | 0 | 1 | 0 | 4.71E-05 |
| **BRCA2** | 13:32905140 | ACAAAT>A | frameshift_variant | c.771_775delTCAAA | p.Asn257fs | 0 | 3 | 0 | 0.000141403 |
| **BRCA2** | 13:32905146 | CAA>C | frameshift_variant | c.774_775delAA | p.Glu260fs | 0 | 1 | 0 | 4.71E-05 |
| **BRCA2** | 13:32906424 | C>A | stop_gained | c.809C>A | p.Ser270* | 0 | 1 | 0 | 4.71E-05 |
| **BRCA2** | 13:32906433 | C>A | stop_gained | c.818C>A | p.Ser273* | 0 | 1 | 0 | 4.71E-05 |
| **BRCA2** | 13:32906507 | AC>A | frameshift_variant | c.893delC | p.Thr298fs | 0 | 1 | 0 | 4.71E-05 |
| **BRCA2** | 13:32906529 | AAGAT>A | frameshift_variant | c.917_920delATAG | p.Asp306fs | 0 | 6 | 0 | 0.000282805 |
| **BRCA2** | 13:32906565 | C>CA | frameshift_variant | c.956dupA | p.Asn319fs | 0 | 1 | 0 | 4.71E-05 |
| **BRCA2** | 13:32906576 | C>T | stop_gained | c.961C>T | p.Gln321* | 0 | 1 | 0 | 4.71E-05 |
| **BRCA2** | 13:32906602 | GA>G | frameshift_variant | c.994delA | p.Ile332fs | 0 | 3 | 0 | 0.000141403 |
| **BRCA2** | 13:32906654 | C>T | stop_gained | c.1039C>T | p.Gln347* | 0 | 1 | 0 | 4.71E-05 |
| **BRCA2** | 13:32906663 | GA>G | frameshift_variant | c.1053delA | p.Lys351fs | 0 | 1 | 0 | 4.71E-05 |
| **BRCA2** | 13:32906737 | GC>G | frameshift_variant | c.1125delC | p.Phe376fs | 0 | 1 | 0 | 4.71E-05 |
| **BRCA2** | 13:32906848 | AC>A | frameshift_variant | c.1237delC | p.Leu413fs | 0 | 2 | 0 | 9.43E-05 |
| **BRCA2** | 13:32906915 | AAAAG>A | frameshift_variant | c.1310_1313delAAGA | p.Lys437fs | 0 | 3 | 0 | 0.000141403 |
| **BRCA2** | 13:32906981 | G>T | stop_gained | c.1366G>T | p.Glu456* | 0 | 1 | 0 | 4.71E-05 |
| **BRCA2** | 13:32907003 | CAG>C | frameshift_variant | c.1389_1390delAG | p.Val464fs | 0 | 1 | 0 | 4.71E-05 |
| **BRCA2** | 13:32907014 | A>T | stop_gained | c.1399A>T | p.Lys467* | 2 | 1 | 0.000310849 | 4.71E-05 |
| **BRCA2** | 13:32907042 | CTCAT>C | frameshift_variant | c.1428_1431delTCAT | p.His477fs | 0 | 1 | 0 | 4.71E-05 |
| **BRCA2** | 13:32907065 | G>GA | frameshift_variant | c.1450_1451insA | p.Val484fs | 0 | 1 | 0 | 4.71E-05 |
| **BRCA2** | 13:32907070 | GC>G | frameshift_variant | c.1456delC | p.Gln486fs | 0 | 1 | 0 | 4.71E-05 |
| **BRCA2** | 13:32907139 | AAG>A | frameshift_variant | c.1528_1529delGA | p.Glu510fs | 0 | 1 | 0 | 4.71E-05 |
| **BRCA2** | 13:32907171 | GT>G | frameshift_variant | c.1561delT | p.Ser521fs | 0 | 1 | 0 | 4.71E-05 |
| **BRCA2** | 13:32907209 | G>T | stop_gained | c.1594G>T | p.Glu532* | 0 | 1 | 0 | 4.71E-05 |
| **BRCA2** | 13:32907260 | A>T | stop_gained | c.1645A>T | p.Lys549* | 0 | 1 | 0 | 4.71E-05 |
| **BRCA2** | 13:32907292 | TA>T | frameshift_variant | c.1679delA | p.Asn560fs | 0 | 1 | 0 | 4.71E-05 |
| **BRCA2** | 13:32907375 | CAAAT>C | frameshift_variant | c.1763_1766delATAA | p.Asn588fs | 0 | 2 | 0 | 9.43E-05 |
| **BRCA2** | 13:32907379 | TAA>T | frameshift_variant | c.1765_1766delAA | p.Lys589fs | 0 | 3 | 0 | 0.000141403 |
| **BRCA2** | 13:32907382 | GTTTA>G | frameshift_variant | c.1773_1776delTTAT | p.Ile591fs | 0 | 3 | 0 | 0.000141403 |
| **BRCA2** | 13:32907408 | CATCTT>C | frameshift_variant | c.1796_1800delCTTAT | p.Ser599fs | 0 | 3 | 0 | 0.000141403 |
| **BRCA2** | 13:32907420 | G>GA | frameshift_variant | c.1813dupA | p.Ile605fs | 0 | 2 | 0 | 9.43E-05 |
| **BRCA2** | 13:32907420 | GA>G | frameshift_variant | c.1813delA | p.Ile605fs | 0 | 1 | 0 | 4.71E-05 |
| **BRCA2** | 13:32907455 | ATT>A | frameshift_variant | c.1841_1842delTT | p.Ile614fs | 0 | 1 | 0 | 4.71E-05 |
| **BRCA2** | 13:32907455 | ATTAACTG>A | frameshift_variant | c.1843_1849delAACTGTT | p.Asn615fs | 0 | 1 | 0 | 4.71E-05 |
| **BRCA2** | 13:32907502 | T>TA | frameshift_variant | c.1888dupA | p.Thr630fs | 0 | 1 | 0 | 4.71E-05 |
| **BRCA2** | 13:32907502 | T>TAA | frameshift_variant | c.1888_1889insAA | p.Thr630fs | 0 | 1 | 0 | 4.71E-05 |
| **BRCA2** | 13:32910516 | CAT>C | frameshift_variant | c.2025_2026delAT | p.Cys676fs | 0 | 2 | 0 | 9.43E-05 |
| **BRCA2** | 13:32910550 | TGATTA>T | frameshift_variant | c.2059_2063delGATTA | p.Asp687fs | 0 | 9 | 0 | 0.000424208 |
| **BRCA2** | 13:32910556 | T>A | stop_gained | c.2064T>A | p.Tyr688* | 3 | 0 | 0.000466273 | 0 |
| **BRCA2** | 13:32910587 | C>T | stop_gained | c.2095C>T | p.Gln699* | 0 | 1 | 0 | 4.71E-05 |
| **BRCA2** | 13:32910661 | CA>C | frameshift_variant | c.2175delA | p.Val726fs | 0 | 1 | 0 | 4.71E-05 |
| **BRCA2** | 13:32910723 | CAA>C | frameshift_variant | c.2234_2235delAA | p.Lys745fs | 0 | 1 | 0 | 4.71E-05 |
| **BRCA2** | 13:32910742 | T>TA | frameshift_variant | c.2251dupA | p.Thr751fs | 0 | 3 | 0 | 0.000141403 |
| **BRCA2** | 13:32910744 | CT>C | frameshift_variant | c.2253delT | p.Asp752fs | 0 | 1 | 0 | 4.71E-05 |
| **BRCA2** | 13:32910748 | CT>C | frameshift_variant | c.2259delT | p.Gln754fs | 0 | 1 | 0 | 4.71E-05 |
| **BRCA2** | 13:32910767 | CT>C | frameshift_variant | c.2279delT | p.Leu760fs | 0 | 1 | 0 | 4.71E-05 |
| **BRCA2** | 13:32910797 | CT>C | frameshift_variant | c.2307delT | p.Ile770fs | 0 | 1 | 0 | 4.71E-05 |
| **BRCA2** | 13:32910901 | T>G | stop_gained | c.2409T>G | p.Tyr803* | 0 | 1 | 0 | 4.71E-05 |
| **BRCA2** | 13:32910931 | TC>T | frameshift_variant | c.2442delC | p.Met815fs | 0 | 7 | 0 | 0.00032994 |
| **BRCA2** | 13:32910963 | T>G | stop_gained | c.2471T>G | p.Leu824* | 0 | 5 | 0 | 0.000235671 |
| **BRCA2** | 13:32911016 | GT>G | frameshift_variant | c.2525delT | p.Val842fs | 0 | 1 | 0 | 4.71E-05 |
| **BRCA2** | 13:32911064 | A>T | stop_gained | c.2572A>T | p.Arg858* | 1 | 0 | 0.000155424 | 0 |
| **BRCA2** | 13:32911085 | G>T | stop_gained | c.2593G>T | p.Glu865* | 0 | 1 | 0 | 4.71E-05 |
| **BRCA2** | 13:32911142 | TCAGA>T | frameshift_variant | c.2653_2656delGACA | p.Asp885fs | 0 | 1 | 0 | 4.71E-05 |
| **BRCA2** | 13:32911242 | T>TA | frameshift_variant | c.2753dupA | p.Asn918fs | 0 | 2 | 0 | 9.43E-05 |
| **BRCA2** | 13:32911297 | TAAAC>T | frameshift_variant | c.2808_2811delACAA | p.Ala938fs | 0 | 13 | 0 | 0.000612745 |
| **BRCA2** | 13:32911317 | CAATT>C | frameshift_variant | c.2828_2831delTTAA | p.Ile943fs | 0 | 1 | 0 | 4.71E-05 |
| **BRCA2** | 13:32911317 | C>G | stop_gained | c.2825C>G | p.Ser942* | 1 | 0 | 0.000155424 | 0 |
| **BRCA2** | 13:32911334 | GT>G | frameshift_variant | c.2845delT | p.Tyr949fs | 0 | 1 | 0 | 4.71E-05 |
| **BRCA2** | 13:32911406 | A>T | stop_gained | c.2914A>T | p.Lys972* | 0 | 1 | 0 | 4.71E-05 |
| **BRCA2** | 13:32911410 | C>A | stop_gained | c.2918C>A | p.Ser973* | 0 | 1 | 0 | 4.71E-05 |
| **BRCA2** | 13:32911441 | A>AG | frameshift_variant | c.2950dupG | p.Glu984fs | 0 | 1 | 0 | 4.71E-05 |
| **BRCA2** | 13:32911442 | GA>G | frameshift_variant | c.2957delA | p.Asn986fs | 2 | 3 | 0.000310849 | 0.000141403 |
| **BRCA2** | 13:32911442 | G>GA | frameshift_variant | c.2957dupA | p.Asn986fs | 0 | 2 | 0 | 9.43E-05 |
| **BRCA2** | 13:32911482 | T>G | stop_gained | c.2990T>G | p.Leu997* | 0 | 1 | 0 | 4.71E-05 |
| **BRCA2** | 13:32911494 | C>G | stop_gained | c.3002C>G | p.Ser1001* | 1 | 1 | 0.000155424 | 4.71E-05 |
| **BRCA2** | 13:32911582 | CT>C | frameshift_variant | c.3092delT | p.Phe1031fs | 0 | 1 | 0 | 4.71E-05 |
| **BRCA2** | 13:32911598 | GA>G | frameshift_variant | c.3108delA | p.Glu1036fs | 0 | 1 | 0 | 4.71E-05 |
| **BRCA2** | 13:32911601 | C>T | stop_gained | c.3109C>T | p.Gln1037* | 1 | 20 | 0.000155424 | 0.000942685 |
| **BRCA2** | 13:32911647 | C>CAA | frameshift_variant | c.3156_3157insAA | p.Leu1053fs | 0 | 1 | 0 | 4.71E-05 |
| **BRCA2** | 13:32911679 | C>T | stop_gained | c.3187C>T | p.Gln1063* | 0 | 1 | 0 | 4.71E-05 |
| **BRCA2** | 13:32911683 | CAATT>C | frameshift_variant | c.3195_3198delTAAT | p.Asn1066fs | 0 | 3 | 0 | 0.000141403 |
| **BRCA2** | 13:32911709 | C>T | stop_gained | c.3217C>T | p.Gln1073* | 0 | 4 | 0 | 0.000188537 |
| **BRCA2** | 13:32911735 | T>TA | frameshift_variant | c.3248dupA | p.Asn1083fs | 0 | 1 | 0 | 4.71E-05 |
| **BRCA2** | 13:32911747 | T>TA | frameshift_variant | c.3256dupA | p.Ile1086fs | 0 | 1 | 0 | 4.71E-05 |
| **BRCA2** | 13:32911775 | C>T | stop_gained | c.3283C>T | p.Gln1095* | 0 | 1 | 0 | 4.71E-05 |
| **BRCA2** | 13:32911854 | C>G | stop_gained | c.3362C>G | p.Ser1121* | 0 | 1 | 0 | 4.71E-05 |
| **BRCA2** | 13:32911855 | AG>A | frameshift_variant | c.3365delG | p.Gly1122fs | 0 | 2 | 0 | 9.43E-05 |
| **BRCA2** | 13:32911877 | C>T | stop_gained | c.3385C>T | p.Gln1129* | 0 | 1 | 0 | 4.71E-05 |
| **BRCA2** | 13:32912032 | GC>G | frameshift_variant | c.3541delC | p.Gln1181fs | 1 | 1 | 0.000155424 | 4.71E-05 |
| **BRCA2** | 13:32912089 | CTG>C | frameshift_variant | c.3599_3600delGT | p.Cys1200fs | 0 | 1 | 0 | 4.71E-05 |
| **BRCA2** | 13:32912175 | ATG>A | frameshift_variant | c.3685_3686delGT | p.Val1229fs | 0 | 1 | 0 | 4.71E-05 |
| **BRCA2** | 13:32912195 | C>T | stop_gained | c.3703C>T | p.Gln1235* | 0 | 2 | 0 | 9.43E-05 |
| **BRCA2** | 13:32912210 | CTG>C | frameshift_variant | c.3720_3721delGT | p.Phe1241fs | 0 | 1 | 0 | 4.71E-05 |
| **BRCA2** | 13:32912231 | AT>A | frameshift_variant | c.3741delT | p.Ser1248fs | 0 | 1 | 0 | 4.71E-05 |
| **BRCA2** | 13:32912233 | TAGTG>T | frameshift_variant | c.3744_3747delTGAG | p.Ser1248fs | 0 | 2 | 0 | 9.43E-05 |
| **BRCA2** | 13:32912287 | T>A | stop_gained | c.3795T>A | p.Cys1265* | 0 | 1 | 0 | 4.71E-05 |
| **BRCA2** | 13:32912337 | CTG>C | frameshift_variant | c.3847_3848delGT | p.Val1283fs | 2 | 6 | 0.000310849 | 0.000282805 |
| **BRCA2** | 13:32912345 | GA>G | frameshift_variant | c.3860delA | p.Asn1287fs | 0 | 1 | 0 | 4.71E-05 |
| **BRCA2** | 13:32912353 | TAATA>T | frameshift_variant | c.3865_3868delAAAT | p.Lys1289fs | 0 | 2 | 0 | 9.43E-05 |
| **BRCA2** | 13:32912375 | C>T | stop_gained | c.3883C>T | p.Gln1295* | 0 | 2 | 0 | 9.43E-05 |
| **BRCA2** | 13:32912414 | G>T | stop_gained | c.3922G>T | p.Glu1308* | 0 | 2 | 0 | 9.43E-05 |
| **BRCA2** | 13:32912528 | ACT>A | frameshift_variant | c.4037_4038delCT | p.Thr1346fs | 0 | 1 | 0 | 4.71E-05 |
| **BRCA2** | 13:32912581 | CAT>C | frameshift_variant | c.4092_4093delAT | p.Ile1364fs | 0 | 1 | 0 | 4.71E-05 |
| **BRCA2** | 13:32912612 | A>T | stop_gained | c.4120A>T | p.Lys1374* | 0 | 1 | 0 | 4.71E-05 |
| **BRCA2** | 13:32912622 | ACACT>A | frameshift_variant | c.4133_4136delCTCA | p.Thr1378fs | 0 | 1 | 0 | 4.71E-05 |
| **BRCA2** | 13:32912855 | G>T | stop_gained | c.4363G>T | p.Glu1455* | 1 | 0 | 0.000155424 | 0 |
| **BRCA2** | 13:32912899 | CATAAG>C | frameshift_variant | c.4409_4413delTAAGA | p.Ile1470fs | 0 | 1 | 0 | 4.71E-05 |
| **BRCA2** | 13:32912901 | TAAGA>T | frameshift_variant | c.4415_4418delAGAA | p.Lys1472fs | 0 | 3 | 0 | 0.000141403 |
| **BRCA2** | 13:32912958 | AAATAC>A | frameshift_variant | c.4467_4471delAATAC | p.Lys1489fs | 0 | 2 | 0 | 9.43E-05 |
| **BRCA2** | 13:32912966 | A>T | stop_gained | c.4474A>T | p.Lys1492* | 0 | 1 | 0 | 4.71E-05 |
| **BRCA2** | 13:32913032 | GA>G | frameshift_variant | c.4544delA | p.Lys1515fs | 0 | 2 | 0 | 9.43E-05 |
| **BRCA2** | 13:32913101 | GA>G | frameshift_variant | c.4611delA | p.Glu1537fs | 0 | 2 | 0 | 9.43E-05 |
| **BRCA2** | 13:32913105 | CTT>C | frameshift_variant | c.4615_4616delTT | p.Leu1539fs | 0 | 1 | 0 | 4.71E-05 |
| **BRCA2** | 13:32913138 | AAG>A | frameshift_variant | c.4649_4650delAG | p.Glu1550fs | 0 | 1 | 0 | 4.71E-05 |
| **BRCA2** | 13:32913277 | GA>G | frameshift_variant | c.4787delA | p.Asn1596fs | 0 | 1 | 0 | 4.71E-05 |
| **BRCA2** | 13:32913365 | G>GA | frameshift_variant | c.4877dupA | p.Asn1626fs | 0 | 2 | 0 | 9.43E-05 |
| **BRCA2** | 13:32913403 | T>TA | frameshift_variant | c.4914dupA | p.Val1639fs | 0 | 2 | 0 | 9.43E-05 |
| **BRCA2** | 13:32913417 | ATG>A | frameshift_variant | c.4927_4928delGT | p.Val1643fs | 0 | 1 | 0 | 4.71E-05 |
| **BRCA2** | 13:32913424 | AAAAG>A | frameshift_variant | c.4936_4939delGAAA | p.Glu1646fs | 0 | 1 | 0 | 4.71E-05 |
| **BRCA2** | 13:32913531 | CTG>C | frameshift_variant | c.5042_5043delTG | p.Val1681fs | 0 | 3 | 0 | 0.000141403 |
| **BRCA2** | 13:32913619 | TTATG>T | frameshift_variant | c.5130_5133delTGTA | p.Tyr1710fs | 0 | 1 | 0 | 4.71E-05 |
| **BRCA2** | 13:32913635 | TTGTA>T | frameshift_variant | c.5146_5149delTATG | p.Tyr1716fs | 0 | 1 | 0 | 4.71E-05 |
| **BRCA2** | 13:32913650 | TCAAA>T | frameshift_variant | c.5161_5164delAACA | p.Asn1721fs | 0 | 1 | 0 | 4.71E-05 |
| **BRCA2** | 13:32913655 | CAG>C | frameshift_variant | c.5164_5165delAG | p.Ser1722fs | 1 | 13 | 0.000155424 | 0.000612745 |
| **BRCA2** | 13:32913657 | G>GT | frameshift_variant | c.5166dupT | p.Thr1723fs | 0 | 5 | 0 | 0.000235671 |
| **BRCA2** | 13:32913777 | ATC>A | frameshift_variant | c.5290_5291delTC | p.Ser1764fs | 0 | 1 | 0 | 4.71E-05 |
| **BRCA2** | 13:32913823 | G>GA | frameshift_variant | c.5333dupA | p.Asn1778fs | 0 | 2 | 0 | 9.43E-05 |
| **BRCA2** | 13:32913952 | C>A | stop_gained | c.5460C>A | p.Cys1820* | 1 | 3 | 0.000155424 | 0.000141403 |
| **BRCA2** | 13:32913959 | A>T | stop_gained | c.5467A>T | p.Lys1823* | 0 | 2 | 0 | 9.43E-05 |
| **BRCA2** | 13:32913994 | T>TA | frameshift_variant | c.5504dupA | p.Asn1835fs | 0 | 2 | 0 | 9.43E-05 |
| **BRCA2** | 13:32914046 | G>GT | frameshift_variant | c.5557dupT | p.Cys1853fs | 0 | 1 | 0 | 4.71E-05 |
| **BRCA2** | 13:32914065 | CAATT>C | frameshift_variant | c.5576_5579delTTAA | p.Ile1859fs | 0 | 7 | 0 | 0.00032994 |
| **BRCA2** | 13:32914070 | A>T | stop_gained | c.5578A>T | p.Lys1860* | 0 | 2 | 0 | 9.43E-05 |
| **BRCA2** | 13:32914108 | AGTAAT>A | frameshift_variant | c.5617_5621delGTAAT | p.Val1873fs | 0 | 2 | 0 | 9.43E-05 |
| **BRCA2** | 13:32914113 | T>TA | frameshift_variant | c.5621_5622insA | p.Lys1875fs | 0 | 1 | 0 | 4.71E-05 |
| **BRCA2** | 13:32914115 | A>T | stop_gained | c.5623A>T | p.Lys1875* | 0 | 1 | 0 | 4.71E-05 |
| **BRCA2** | 13:32914123 | CA>C | frameshift_variant | c.5633delA | p.Asn1878fs | 0 | 1 | 0 | 4.71E-05 |
| **BRCA2** | 13:32914137 | C>A | stop_gained | c.5645C>A | p.Ser1882* | 0 | 8 | 0 | 0.000377074 |
| **BRCA2** | 13:32914172 | T>TA | frameshift_variant | c.5681dupA | p.Tyr1894fs | 0 | 3 | 0 | 0.000141403 |
| **BRCA2** | 13:32914174 | C>A | stop_gained | c.5682C>A | p.Tyr1894* | 0 | 4 | 0 | 0.000188537 |
| **BRCA2** | 13:32914174 | C>G | stop_gained | c.5682C>G | p.Tyr1894* | 2 | 32 | 0.000310849 | 0.001508296 |
| **BRCA2** | 13:32914209 | ACT>A | frameshift_variant | c.5722_5723delCT | p.Leu1908fs | 0 | 10 | 0 | 0.000471342 |
| **BRCA2** | 13:32914219 | TA>T | frameshift_variant | c.5729delA | p.Asn1910fs | 0 | 1 | 0 | 4.71E-05 |
| **BRCA2** | 13:32914242 | C>G | stop_gained | c.5750C>G | p.Ser1917* | 0 | 2 | 0 | 9.43E-05 |
| **BRCA2** | 13:32914246 | TA>T | frameshift_variant | c.5756delA | p.Lys1919fs | 0 | 1 | 0 | 4.71E-05 |
| **BRCA2** | 13:32914260 | ACATT>A | frameshift_variant | c.5771_5774delTTCA | p.Ile1924fs | 0 | 2 | 0 | 9.43E-05 |
| **BRCA2** | 13:32914283 | C>T | stop_gained | c.5791C>T | p.Gln1931* | 0 | 2 | 0 | 9.43E-05 |
| **BRCA2** | 13:32914339 | TGTTA>T | frameshift_variant | c.5851_5854delAGTT | p.Ser1951fs | 0 | 1 | 0 | 4.71E-05 |
| **BRCA2** | 13:32914356 | C>G | stop_gained | c.5864C>G | p.Ser1955* | 0 | 3 | 0 | 0.000141403 |
| **BRCA2** | 13:32914451 | C>T | stop_gained | c.5959C>T | p.Gln1987* | 1 | 4 | 0.000155424 | 0.000188537 |
| **BRCA2** | 13:32914540 | T>TA | frameshift_variant | c.6052dupA | p.Ser2018fs | 0 | 2 | 0 | 9.43E-05 |
| **BRCA2** | 13:32914569 | CAAGAG>C | frameshift_variant | c.6082_6086delGAAGA | p.Glu2028fs | 0 | 1 | 0 | 4.71E-05 |
| **BRCA2** | 13:32914587 | C>CT | frameshift_variant | c.6096dupT | p.Ile2033fs | 0 | 1 | 0 | 4.71E-05 |
| **BRCA2** | 13:32914647 | C>G | stop_gained | c.6155C>G | p.Ser2052* | 0 | 2 | 0 | 9.43E-05 |
| **BRCA2** | 13:32914694 | A>AT | frameshift_variant | c.6206dupT | p.Leu2069fs | 0 | 1 | 0 | 4.71E-05 |
| **BRCA2** | 13:32914716 | AAGTT>A | frameshift_variant | c.6226_6229delGTTA | p.Val2076fs | 0 | 1 | 0 | 4.71E-05 |
| **BRCA2** | 13:32914736 | GA>G | frameshift_variant | c.6246delA | p.Glu2082fs | 0 | 1 | 0 | 4.71E-05 |
| **BRCA2** | 13:32914746 | TAATC>T | frameshift_variant | c.6256_6259delATCA | p.Ile2086fs | 0 | 1 | 0 | 4.71E-05 |
| **BRCA2** | 13:32914795 | TG>T | frameshift_variant | c.6304delG | p.Val2102fs | 0 | 6 | 0 | 0.000282805 |
| **BRCA2** | 13:32914825 | G>GA | frameshift_variant | c.6334dupA | p.Arg2112fs | 0 | 1 | 0 | 4.71E-05 |
| **BRCA2** | 13:32914851 | C>G | stop_gained | c.6359C>G | p.Ser2120* | 0 | 1 | 0 | 4.71E-05 |
| **BRCA2** | 13:32914877 | G>T | stop_gained | c.6385G>T | p.Glu2129* | 0 | 2 | 0 | 9.43E-05 |
| **BRCA2** | 13:32914885 | A>AAT | frameshift_variant | c.6393_6394insAT | p.Leu2132fs | 1 | 2 | 0.000155424 | 9.43E-05 |
| **BRCA2** | 13:32914893 | ATAACT>A | frameshift_variant | c.6405_6409delCTTAA | p.Asn2135fs | 1 | 5 | 0.000155424 | 0.000235671 |
| **BRCA2** | 13:32914897 | CTTAAATG>C | frameshift_variant | c.6408_6414delAAATGTT | p.Asn2137fs | 0 | 1 | 0 | 4.71E-05 |
| **BRCA2** | 13:32914899 | T>G | stop_gained | c.6407T>G | p.Leu2136* | 0 | 1 | 0 | 4.71E-05 |
| **BRCA2** | 13:32914922 | G>GA | frameshift_variant | c.6434dupA | p.Asn2145fs | 0 | 1 | 0 | 4.71E-05 |
| **BRCA2** | 13:32914932 | ACT>A | frameshift_variant | c.6443_6444delCT | p.Ser2148fs | 0 | 1 | 0 | 4.71E-05 |
| **BRCA2** | 13:32914937 | AT>A | frameshift_variant | c.6447delT | p.Val2151fs | 0 | 1 | 0 | 4.71E-05 |
| **BRCA2** | 13:32914938 | T>TTA | frameshift_variant | c.6447_6448dupTA | p.Lys2150fs | 0 | 4 | 0 | 0.000188537 |
| **BRCA2** | 13:32914939 | T>TA | frameshift_variant | c.6450dupA | p.Val2151fs | 0 | 2 | 0 | 9.43E-05 |
| **BRCA2** | 13:32914953 | ATC>A | frameshift_variant | c.6468_6469delTC | p.Gln2157fs | 1 | 7 | 0.000155424 | 0.00032994 |
| **BRCA2** | 13:32914953 | ATCTC>A | frameshift_variant | c.6466_6469delTCTC | p.Ser2156fs | 0 | 4 | 0 | 0.000188537 |
| **BRCA2** | 13:32914973 | GACAA>G | frameshift_variant | c.6486_6489delACAA | p.Lys2162fs | 0 | 5 | 0 | 0.000235671 |
| **BRCA2** | 13:32915002 | AGT>A | frameshift_variant | c.6513_6514delGT | p.Ser2172fs | 1 | 1 | 0.000155424 | 4.71E-05 |
| **BRCA2** | 13:32915009 | C>CT | frameshift_variant | c.6519dupT | p.Val2174fs | 0 | 1 | 0 | 4.71E-05 |
| **BRCA2** | 13:32915038 | AG>A | frameshift_variant | c.6547delG | p.Glu2183fs | 0 | 4 | 0 | 0.000188537 |
| **BRCA2** | 13:32915039 | G>T | stop_gained | c.6547G>T | p.Glu2183* | 0 | 2 | 0 | 9.43E-05 |
| **BRCA2** | 13:32915041 | AC>A | frameshift_variant | c.6550delC | p.Gln2184fs | 0 | 1 | 0 | 4.71E-05 |
| **BRCA2** | 13:32915043 | AG>A | frameshift_variant | c.6553delG | p.Ala2185fs | 0 | 1 | 0 | 4.71E-05 |
| **BRCA2** | 13:32915082 | CTG>C | frameshift_variant | c.6591_6592delTG | p.Glu2198fs | 0 | 10 | 0 | 0.000471342 |
| **BRCA2** | 13:32915112 | CA>C | frameshift_variant | c.6623delA | p.Asn2208fs | 0 | 3 | 0 | 0.000141403 |
| **BRCA2** | 13:32915112 | CAA>C | frameshift_variant | c.6622_6623delAA | p.Asn2208fs | 0 | 1 | 0 | 4.71E-05 |
| **BRCA2** | 13:32915190 | C>CTTTT | frameshift_variant | c.6699_6702dupTTTT | p.Met2235fs | 0 | 2 | 0 | 9.43E-05 |
| **BRCA2** | 13:32915249 | CTT>C | frameshift_variant | c.6761_6762delTT | p.Phe2254fs | 0 | 1 | 0 | 4.71E-05 |
| **BRCA2** | 13:32915292 | C>A | stop_gained | c.6800C>A | p.Ser2267* | 0 | 2 | 0 | 9.43E-05 |
| **BRCA2** | 13:32920963 | G>A | splice_acceptor_variant | c.6938-1G>A |  | 0 | 1 | 0 | 4.71E-05 |
| **BRCA2** | 13:32920978 | C>T | stop_gained | c.6952C>T | p.Arg2318* | 0 | 1 | 0 | 4.71E-05 |
| **BRCA2** | 13:32929030 | CA>C | frameshift_variant | c.7043delA | p.Asn2348fs | 1 | 3 | 0.000155424 | 0.000141403 |
| **BRCA2** | 13:32929033 | A>AT | frameshift_variant | c.7047dupT | p.Thr2350fs | 0 | 2 | 0 | 9.43E-05 |
| **BRCA2** | 13:32929093 | T>A | stop_gained | c.7103T>A | p.Leu2368* | 1 | 0 | 0.000155424 | 0 |
| **BRCA2** | 13:32929123 | C>G | stop_gained | c.7133C>G | p.Ser2378* | 0 | 10 | 0 | 0.000471342 |
| **BRCA2** | 13:32929130 | TC>T | frameshift_variant | c.7142delC | p.Pro2381fs | 0 | 1 | 0 | 4.71E-05 |
| **BRCA2** | 13:32929161 | G>GA | frameshift_variant | c.7177dupA | p.Met2393fs | 0 | 1 | 0 | 4.71E-05 |
| **BRCA2** | 13:32929193 | AC>A | frameshift_variant | c.7205delC | p.Pro2402fs | 1 | 0 | 0.000155424 | 0 |
| **BRCA2** | 13:32929205 | CTT>C | frameshift_variant | c.7217_7218delTT | p.Phe2406fs | 0 | 1 | 0 | 4.71E-05 |
| **BRCA2** | 13:32929364 | CA>C | frameshift_variant | c.7379delA | p.Asn2460fs | 0 | 1 | 0 | 4.71E-05 |
| **BRCA2** | 13:32929375 | CCAAT>C | frameshift_variant | c.7389_7392delTCAA | p.Asn2463fs | 0 | 1 | 0 | 4.71E-05 |
| **BRCA2** | 13:32929396 | C>CT | frameshift_variant | c.7409dupT | p.Thr2471fs | 1 | 3 | 0.000155424 | 0.000141403 |
| **BRCA2** | 13:32929396 | CTT>C | frameshift_variant | c.7408_7409delTT | p.Phe2470fs | 0 | 2 | 0 | 9.43E-05 |
| **BRCA2** | 13:32930609 | C>T | stop_gained | c.7480C>T | p.Arg2494* | 0 | 2 | 0 | 9.43E-05 |
| **BRCA2** | 13:32930628 | G>GGCAAC | frameshift_variant | c.7502_7506dupAACGC | p.Val2503fs | 0 | 1 | 0 | 4.71E-05 |
| **BRCA2** | 13:32930638 | CT>C | frameshift_variant | c.7512delT | p.Pro2505fs | 0 | 1 | 0 | 4.71E-05 |
| **BRCA2** | 13:32930645 | C>T | stop_gained | c.7516C>T | p.Gln2506* | 0 | 1 | 0 | 4.71E-05 |
| **BRCA2** | 13:32930648 | CCAGG>C | frameshift_variant | c.7523_7526delGCAG | p.Gly2508fs | 0 | 1 | 0 | 4.71E-05 |
| **BRCA2** | 13:32930687 | C>T | stop_gained | c.7558C>T | p.Arg2520* | 0 | 1 | 0 | 4.71E-05 |
| **BRCA2** | 13:32930728 | TG>T | frameshift_variant | c.7600delG | p.Ala2534fs | 0 | 1 | 0 | 4.71E-05 |
| **BRCA2** | 13:32930747 | G>A | splice_donor_variant | c.7617+1G>A |  | 0 | 2 | 0 | 9.43E-05 |
| **BRCA2** | 13:32931884 | T>TAC | frameshift_variant | c.7624_7625dupAC | p.Tyr2543fs | 0 | 1 | 0 | 4.71E-05 |
| **BRCA2** | 13:32931890 | TG>T | frameshift_variant | c.7631delG | p.Gly2544fs | 0 | 1 | 0 | 4.71E-05 |
| **BRCA2** | 13:32931931 | CAG>C | frameshift_variant | c.7673_7674delAG | p.Glu2558fs | 0 | 2 | 0 | 9.43E-05 |
| **BRCA2** | 13:32931937 | C>CT | frameshift_variant | c.7680dupT | p.Gln2561fs | 0 | 3 | 0 | 0.000141403 |
| **BRCA2** | 13:32936710 | G>A | stop_gained | c.7856G>A | p.Trp2619* | 0 | 1 | 0 | 4.71E-05 |
| **BRCA2** | 13:32936731 | G>A | stop_gained | c.7877G>A | p.Trp2626* | 0 | 1 | 0 | 4.71E-05 |
| **BRCA2** | 13:32936732 | G>C | missense_variant | c.7878G>C | p.Trp2626Cys | 0 | 1 | 0 | 4.71E-05 |
| **BRCA2** | 13:32936741 | GAA>G | frameshift_variant | c.7889_7890delAA | p.Lys2630fs | 0 | 1 | 0 | 4.71E-05 |
| **BRCA2** | 13:32936741 | G>A | stop_gained | c.7887G>A | p.Trp2629* | 0 | 1 | 0 | 4.71E-05 |
| **BRCA2** | 13:32936821 | T>TA | frameshift_variant | c.7971dupA | p.Tyr2658fs | 0 | 1 | 0 | 4.71E-05 |
| **BRCA2** | 13:32937314 | A>G | splice_acceptor_variant | c.7977-2A>G |  | 0 | 1 | 0 | 4.71E-05 |
| **BRCA2** | 13:32937315 | G>T | splice_acceptor_variant | c.7977-1G>T |  | 0 | 1 | 0 | 4.71E-05 |
| **BRCA2** | 13:32937348 | C>T | missense_variant | c.8009C>T | p.Ser2670Leu | 0 | 1 | 0 | 4.71E-05 |
| **BRCA2** | 13:32937354 | T>TA | frameshift_variant | c.8021dupA | p.Ile2675fs | 0 | 1 | 0 | 4.71E-05 |
| **BRCA2** | 13:32937403 | CTG>C | frameshift_variant | c.8068_8069delGT | p.Val2690fs | 0 | 1 | 0 | 4.71E-05 |
| **BRCA2** | 13:32937507 | A>G | missense_variant | c.8168A>G | p.Asp2723Gly | 1 | 0 | 0.000155424 | 0 |
| **BRCA2** | 13:32937508 | TG>T | frameshift_variant | c.8172delG | p.Trp2725fs | 0 | 3 | 0 | 0.000141403 |
| **BRCA2** | 13:32937551 | GC>G | frameshift_variant | c.8213delC | p.Ala2738fs | 0 | 1 | 0 | 4.71E-05 |
| **BRCA2** | 13:32937671 | G>A | splice_donor_variant | c.8331+1G>A |  | 0 | 1 | 0 | 4.71E-05 |
| **BRCA2** | 13:32944538 | G>T | splice_acceptor_variant | c.8332-1G>T |  | 0 | 1 | 0 | 4.71E-05 |
| **BRCA2** | 13:32944539 | AT>A | frameshift_variant | c.8335delT | p.Ser2779fs | 0 | 1 | 0 | 4.71E-05 |
| **BRCA2** | 13:32944606 | CTTTT>C | frameshift_variant | c.8400_8403delTTTT | p.Phe2801fs | 1 | 3 | 0.000155424 | 0.000141403 |
| **BRCA2** | 13:32944606 | C>CA | frameshift_variant | c.8399_8400insA | p.Pro2802fs | 0 | 10 | 0 | 0.000471342 |
| **BRCA2** | 13:32944684 | AC>A | frameshift_variant | c.8480delC | p.Pro2827fs | 0 | 1 | 0 | 4.71E-05 |
| **BRCA2** | 13:32944685 | C>A | stop_gained | c.8478C>A | p.Tyr2826* | 0 | 1 | 0 | 4.71E-05 |
| **BRCA2** | 13:32944692 | C>T | stop_gained | c.8485C>T | p.Gln2829* | 1 | 8 | 0.000155424 | 0.000377074 |
| **BRCA2** | 13:32945092 | G>A | splice_acceptor_variant | c.8488-1G>A |  | 0 | 1 | 0 | 4.71E-05 |
| **BRCA2** | 13:32945095 | G>A | stop_gained | c.8490G>A | p.Trp2830* | 0 | 1 | 0 | 4.71E-05 |
| **BRCA2** | 13:32945109 | C>A | stop_gained | c.8504C>A | p.Ser2835* | 0 | 3 | 0 | 0.000141403 |
| **BRCA2** | 13:32945109 | C>G | stop_gained | c.8504C>G | p.Ser2835* | 0 | 1 | 0 | 4.71E-05 |
| **BRCA2** | 13:32945122 | C>A | stop_gained | c.8517C>A | p.Tyr2839* | 0 | 1 | 0 | 4.71E-05 |
| **BRCA2** | 13:32945149 | AAAGG>A | frameshift_variant | c.8548_8551delGAAG | p.Glu2850fs | 0 | 1 | 0 | 4.71E-05 |
| **BRCA2** | 13:32950922 | C>A | stop_gained | c.8748C>A | p.Tyr2916* | 0 | 1 | 0 | 4.71E-05 |
| **BRCA2** | 13:32953476 | T>A | stop_gained | c.8777T>A | p.Leu2926* | 0 | 2 | 0 | 9.43E-05 |
| **BRCA2** | 13:32953476 | T>TA | frameshift_variant | c.8779dupA | p.Arg2927fs | 0 | 1 | 0 | 4.71E-05 |
| **BRCA2** | 13:32953499 | C>T | stop_gained | c.8800C>T | p.Gln2934* | 1 | 4 | 0.000155424 | 0.000188537 |
| **BRCA2** | 13:32953517 | A>T | stop_gained | c.8818A>T | p.Lys2940* | 0 | 1 | 0 | 4.71E-05 |
| **BRCA2** | 13:32953568 | C>T | stop_gained | c.8869C>T | p.Gln2957* | 0 | 1 | 0 | 4.71E-05 |
| **BRCA2** | 13:32953570 | AAAGGAAC>A | frameshift_variant | c.8876_8882delAACAAGG | p.Glu2959fs | 0 | 1 | 0 | 4.71E-05 |
| **BRCA2** | 13:32953608 | G>A | stop_gained | c.8909G>A | p.Trp2970* | 0 | 1 | 0 | 4.71E-05 |
| **BRCA2** | 13:32953612 | GT>G | frameshift_variant | c.8915delT | p.Leu2972fs | 0 | 5 | 0 | 0.000235671 |
| **BRCA2** | 13:32953612 | GTTGCGTAT>G | frameshift_variant | c.8915_8922delTGCGTATT | p.Leu2972fs | 0 | 2 | 0 | 9.43E-05 |
| **BRCA2** | 13:32953632 | CAA>C | frameshift_variant | c.8939_8940delAA | p.Lys2980fs | 0 | 1 | 0 | 4.71E-05 |
| **BRCA2** | 13:32953642 | AAAAGATTCAGGT>A | frameshift_variant | c.8946_8953+4delAGATTCAGGTAA | p.Lys2982fs | 0 | 1 | 0 | 4.71E-05 |
| **BRCA2** | 13:32953650 | C>G | stop_gained | c.8951C>G | p.Ser2984* | 0 | 5 | 0 | 0.000235671 |
| **BRCA2** | 13:32953653 | G>A | splice_donor_variant | c.8953+1G>A |  | 0 | 1 | 0 | 4.71E-05 |
| **BRCA2** | 13:32953892 | CTGAG>C | frameshift_variant | c.8961_8964delGAGT | p.Ser2988fs | 0 | 1 | 0 | 4.71E-05 |
| **BRCA2** | 13:32953902 | G>A | stop_gained | c.8969G>A | p.Trp2990* | 0 | 2 | 0 | 9.43E-05 |
| **BRCA2** | 13:32953940 | G>T | stop_gained | c.9007G>T | p.Gly3003* | 0 | 4 | 0 | 0.000188537 |
| **BRCA2** | 13:32953968 | CA>C | frameshift_variant | c.9037delA | p.Thr3013fs | 0 | 4 | 0 | 0.000188537 |
| **BRCA2** | 13:32954002 | TAACA>T | frameshift_variant | c.9070_9073delAACA | p.Asn3024fs | 0 | 1 | 0 | 4.71E-05 |
| **BRCA2** | 13:32954004 | ACATACAGTTAG>A | frameshift_variant | c.9074_9084delTACAGTTAGCA | p.Ile3025fs | 0 | 1 | 0 | 4.71E-05 |
| **BRCA2** | 13:32954022 | C>CA | frameshift_variant | c.9097dupA | p.Thr3033fs | 0 | 7 | 0 | 0.00032994 |
| **BRCA2** | 13:32954022 | CA>C | frameshift_variant | c.9097delA | p.Thr3033fs | 0 | 12 | 0 | 0.000565611 |
| **BRCA2** | 13:32954051 | G>A | splice_donor_variant | c.9117+1G>A |  | 0 | 1 | 0 | 4.71E-05 |
| **BRCA2** | 13:32954142 | A>G | splice_acceptor_variant | c.9118-2A>G |  | 0 | 1 | 0 | 4.71E-05 |
| **BRCA2** | 13:32954174 | C>T | stop_gained | c.9148C>T | p.Gln3050* | 0 | 1 | 0 | 4.71E-05 |
| **BRCA2** | 13:32954180 | C>T | missense_variant | c.9154C>T | p.Arg3052Trp | 0 | 3 | 0 | 0.000141403 |
| **BRCA2** | 13:32954263 | TTC>T | frameshift_variant | c.9239_9240delCT | p.Ser3080fs | 0 | 1 | 0 | 4.71E-05 |
| **BRCA2** | 13:32954272 | G>GA | frameshift_variant | c.9253dupA | p.Thr3085fs | 0 | 3 | 0 | 0.000141403 |
| **BRCA2** | 13:32954272 | GA>G | frameshift_variant | c.9253delA | p.Thr3085fs | 0 | 1 | 0 | 4.71E-05 |
| **BRCA2** | 13:32968842 | CTATT>C | frameshift_variant | c.9275_9278delATTT | p.Tyr3092fs | 0 | 3 | 0 | 0.000141403 |
| **BRCA2** | 13:32968842 | CTA>C | frameshift_variant | c.9275_9276delAT | p.Tyr3092fs | 0 | 1 | 0 | 4.71E-05 |
| **BRCA2** | 13:32968863 | C>G | stop_gained | c.9294C>G | p.Tyr3098* | 0 | 3 | 0 | 0.000141403 |
| **BRCA2** | 13:32968898 | A>AT | frameshift_variant | c.9330dupT | p.Glu3111fs | 0 | 7 | 0 | 0.00032994 |
| **BRCA2** | 13:32968925 | T>G | stop_gained | c.9356T>G | p.Leu3119* | 0 | 2 | 0 | 9.43E-05 |
| **BRCA2** | 13:32968937 | G>GC | frameshift_variant | c.9369dupC | p.Asn3124fs | 0 | 1 | 0 | 4.71E-05 |
| **BRCA2** | 13:32968951 | C>T | stop_gained | c.9382C>T | p.Arg3128* | 0 | 6 | 0 | 0.000282805 |
| **BRCA2** | 13:32968967 | C>G | stop_gained | c.9398C>G | p.Ser3133* | 0 | 1 | 0 | 4.71E-05 |
| **BRCA2** | 13:32968968 | AG>A | frameshift_variant | c.9401delG | p.Gly3134fs | 0 | 1 | 0 | 4.71E-05 |
| **BRCA2** | 13:32969004 | GT>G | frameshift_variant | c.9439delT | p.Ser3147fs | 0 | 2 | 0 | 9.43E-05 |
| **BRCA2** | 13:32969071 | G>A | splice_donor_variant | c.9501+1G>A |  | 0 | 1 | 0 | 4.71E-05 |
| **BRCA2** | 13:32971034 | G>A | splice_acceptor_variant | c.9502-1G>A |  | 0 | 2 | 0 | 9.43E-05 |
| **BRCA2** | 13:32971037 | T>TA | frameshift_variant | c.9505dupA | p.Ile3169fs | 0 | 1 | 0 | 4.71E-05 |
| **BRCA2** | 13:32972325 | T>G | stop_gained | c.9675T>G | p.Tyr3225* | 0 | 1 | 0 | 4.71E-05 |
| **BRCA2** | 13:32972342 | C>G | stop_gained | c.9692C>G | p.Ser3231* | 0 | 1 | 0 | 4.71E-05 |
| **BRCA2** | 13:32972402 | AG>A | frameshift_variant | c.9753delG | p.Lys3251fs | 0 | 1 | 0 | 4.71E-05 |
| **BRCA2** | 13:32972445 | CA>C | frameshift_variant | c.9800delA | p.Lys3267fs | 0 | 1 | 0 | 4.71E-05 |
| **BRCA2** | 13:32972533 | C>T | stop_gained | c.9883C>T | p.Gln3295* | 0 | 1 | 0 | 4.71E-05 |

BCs: Breast cancer patients; HCs: Healthy controls;
