# Supplementary material for: Prevalence and reclassification of BRCA1 and BRCA2 variants in a large, unselected Chinese Han breast cancer cohort
Source: J Hematol Oncol. 2021 Jan 18;14:18. doi: 10.1186/s13045-020-01010-0 (PMC7814423; doi:10.1186/s13045-020-01010-0)
Supplement: Supplementary file 6 — Additional file 6: Table S3. Clinical characteristics of BC patients with pathogenic BRCA1/2 variants carriers and non-pathogenic variants carrier. [file 13045_2020_1010_MOESM6_ESM.docx]

**Supplementary Table 3** **Clinical characteristics of** **BC patients with pathogenic *BRCA1/2* variants carriers and non-****pathogenic variants carriers**

| **Characteristics** | | **Numbers of**  **patients** | **Non- pathogenic**  **variants carriers n (%)** | **Pathogenic BRCA1**  **variants carriers n (%)** | **P1-value** | **Pathogenic BRCA2**  **variants carriers n (%)** | **P2-value** |
| --- | --- | --- | --- | --- | --- | --- | --- |
| **Mean age** | 17420 | | 47.94 | 43.05 |  | 44.68 |  |
| **Age, years** | ≦30 | 659 | 3.59% (592/16472) | 10.15% (41/404) | P<0.0001 | 4.78% (26/544) | P<0.0001 |
|  | 30-40 | 3494 | 19.43% (3201/16472) | 32.18% (130/404) |  | 29.96% (163/544) |  |
|  | 40-50 | 6898 | 39.72% (6543/16472) | 36.14% (146/404) |  | 38.42% (209/544) |  |
|  | 50-60 | 4434 | 25.81% (4252/16472) | 17.08% (69/404) |  | 20.77% (113/544) |  |
|  | ＞60 | 1935 | 11.44% (1884/16472) | 4.46% (18/404) |  | 6.07% (33/544) |  |
| **BMI** | <18.5 | 306 | 4.08% (285/6989) | 4.25% (9/212) | P=0.361 | 5.13% (12/234) | P=0.649 |
|  | 18.5-23.9 | 4075 | 54.60% (3816/6989) | 60.85% (129/212) |  | 55.56% (130/234) |  |
|  | ≧24 | 3054 | 41.32% (2888/6989) | 34.91% (74/212) |  | 39.32% (92/234) |  |
| **Histology** | In situ carcinomas | 1051 | 6.30% (1016/16116) | 10.87% (35/322) | P<0.0001 | 11.14% (48/431) | P<0.0001 |
|  | Invasive carcinomas | 1250 | 5.89% (949/16116) | 43.79% (141/322) |  | 37.12% (160/431) |  |
|  | invasive ductalcarcinoma | 14520 | 87.81% (14151/16116) | 45.34% (146/322) |  | 51.74% (223/431) |  |
| **Subtype** | Luminal A | 1507 | 13.43% (1487/11073) | 1.73% (5/289) | P<0.0001 | 4.31% (15/348) | P<0.0001 |
|  | Luminal B | 6310 | 53.81% (5958/11073) | 32.53% (94/289) |  | 74.14% (258/348) |  |
|  | TNBC | 2363 | 19.24% (2130/11073) | 59.86% (173/289) |  | 17.24% (60/348) |  |
|  | HER2 positive | 1530 | 13.53% (1498/11073) | 5.88% (17/289) |  | 4.31% (15/348) |  |
| **Tumor Size** | ≦2 cm | 4932 | 54.10% (4646/8588) | 49.43% (129/261) | P=0.087 | 54.14% (157/290) | P=0.061 |
|  | >2cm, ≦5 cm | 3818 | 41.77% (3587/8588) | 45.21% (118/261) |  | 38.97% (113/290) |  |
|  | >5cm | 389 | 4.13% (355/8588) | 5.36% (14/261) |  | 6.9% (20/290) |  |
| **H****istological grade** | I | 348 | 13.43% (342/2547) | 2.47% (2/81) | P<0.0001 | 4% (4/100) | P=0.008 |
|  | II | 1284 | 46.96% (1196/2547) | 35.8% (29/81) |  | 59% (59/100) |  |
|  | III | 1096 | 39.62% (1009/2547) | 61.73% (50/81) |  | 37% (37/100) |  |
| **Lymph nodes status** | Negative | 5657 | 61.15% (5340/8732) | 68.1% (158/232) | P=0.028 | 56.58% (159/281) | P=0.122 |
|  | Positive | 3588 | 38.85% (3392/8732) | 31.9% (74/232) |  | 43.42% (122/281) |  |
| **Location of cancer** | both sides | 258 | 2.03% (226/11136) | 6.27% (20/319) | P<0.0001 | 3.22% (12/373) | P=0.113 |
|  | one side | 11570 | 97.97% (10910/11136) | 93.73% (299/319) |  | 96.78% (361/373) |  |
| **Family history of BC** | Negative | 15928 | 92.11% (15173/16472) | 76.49% (309/404) | P<0.0001 | 81.99% (446/544) | P<0.0001 |
|  | Positive | 1492 | 7.89% (1299/16472) | 23.51% (95/404) |  | 18.01% (98/544) |  |
| **Family history of**  **other cancers** | Negative | 15686 | 90.36% (14884/16472) | 80.69% (326/404) | P<0.0001 | 87.5% (476/544) | P=0.027 |
|  | Positive | 1734 | 9.64% (1588/16472) | 19.31% (78/404) |  | 12.5% (68/544) |  |

TNBC: Triple-negative breast cancer
