# Supplementary material for: Prevalence and reclassification of BRCA1 and BRCA2 variants in a large, unselected Chinese Han breast cancer cohort
Source: J Hematol Oncol. 2021 Jan 18;14:18. doi: 10.1186/s13045-020-01010-0 (PMC7814423; doi:10.1186/s13045-020-01010-0)
Supplement: Supplementary file 7 — Additional file 7: Table S4. Variants detected in our study whose clinical significance were benign but conflicting interpretations of pathogenicity in ClinVar. [file 13045_2020_1010_MOESM7_ESM.docx]

**Supplementary Table 4 Variants detected in our study whose clinical significance were benign but conflicting interpretations of pathogenicity in ClinVar**

| **Gene** | **hgvs_c** | **hgvs_p** | **Exon** | **BCs frequency** | **HCs frequency** | **p-value** |
| --- | --- | --- | --- | --- | --- | --- |
| **BRCA1** | c.3448C>T | p.Pro1150Ser | 10 | 0.31% (66/21,216) | 0.26% (17/6,434) | 0.638 |
| **BRCA1** | c.2286A>T | p.Arg762Ser | 10 | 0.24% (50/21,216) | 0.19% (12/6,434) | 0.563 |
| **BRCA1** | c.2726A>T | p.Asn909Ile | 10 | 0.21 % (44/21,216) | 0.36% (23/6,434) | 0.046 |
| **BRCA1** | c.3596C>T | p.Ala1199Val | 10 | 0.14% (29/21,216) | 0.08% (5/6,434) | 0.328 |
| **BRCA2** | c.6322C>T | p.Arg2108Cys | 11 | 0.58% (122/21,216) | 0.45% (29/6,434) | 0.279 |
| **BRCA2** | c.4376A>G | p.Asn1459Ser | 11 | 0.21% (45/21,216) | 0.09% (6/6,434) | 0.076 |
| **BRCA2** | c.7088A>G | p.Try2363Cys | 14 | 0.15% (31/21,216) | 0.17% (11/6,434) | 0.791 |

BCs: Breast cancer patients; HCs: Healthy controls;
