# Supplementary material for: Prevalence and reclassification of BRCA1 and BRCA2 variants in a large, unselected Chinese Han breast cancer cohort
Source: J Hematol Oncol. 2021 Jan 18;14:18. doi: 10.1186/s13045-020-01010-0 (PMC7814423; doi:10.1186/s13045-020-01010-0)
Supplement: Supplementary file 8 — Additional file 8: Table S5. 55 VUS detected in our study with distinct status in the Findlay et al. study. [file 13045_2020_1010_MOESM8_ESM.doc]

**Supplementary Table 5 55 VUS detected in our study**

**with distinct** **status in the Findlay et al. study**

| **Gene** | **Chr:posi** | **ref>alt** | **Type** | **hgvs_c** | **hgvs_p** | **Number (BCs)** | **Number (HCs)** | **Annotation** |
| --- | --- | --- | --- | --- | --- | --- | --- | --- |
| **BRCA1** | 17:41276135 | T>C | splice_acceptor_variant | c.-19-3A>G |  | 0 | 4 | Benign |
| **BRCA1** | 17:41276095 | G>A | missense_variant | c.19C>T | p.Arg7Cys | 1 | 1 | Benign |
| **BRCA1** | 17:41276064 | G>C | missense_variant | c.50C>G | p.Ala17Gly | 0 | 1 | Benign |
| **BRCA1** | 17:41276065 | C>A | missense_variant | c.49G>T | p.Ala17Ser | 0 | 1 | Benign |
| **BRCA1** | 17:41276061 | A>T | missense_variant | c.53T>A | p.Met18Lys | 0 | 1 | Pathogenic |
| **BRCA1** | 17:41276061 | A>C | missense_variant | c.53T>G | p.Met18Arg | 0 | 1 | Pathogenic |
| **BRCA1** | 17:41276056 | T>C | missense_variant | c.58A>G | p.Lys20Glu | 0 | 1 | Benign |
| **BRCA1** | 17:41276038 | T>C | missense_variant | c.76A>G | p.Ile26Val | 0 | 1 | Benign |
| **BRCA1** | 17:41267777 | G>A | missense_variant | c.100C>T | p.Pro34Ser | 0 | 1 | Pathogenic |
| **BRCA1** | 17:41267762 | A>C | missense_variant | c.115T>G | p.Cys39Gly | 0 | 1 | Pathogenic |
| **BRCA1** | 17:41267738 | C>T | splice_donor_variant | c.134+5G>A |  | 0 | 1 | Pathogenic |
| **BRCA1** | 17:41258531 | G>A | missense_variant | c.154C>T | p.Leu52Phe | 7 | 10 | Benign |
| **BRCA1** | 17:41258524 | T>C | missense_variant | c.161A>G | p.Gln54Arg | 1 | 1 | Benign |
| **BRCA1** | 17:41258500 | G>A | missense_variant | c.185C>T | p.Pro62Leu | 0 | 1 | Benign |
| **BRCA1** | 17:41258495 | A>G | missense_variant | c.190T>C | p.Cys64Arg | 0 | 2 | Pathogenic |
| **BRCA1** | 17:41258494 | C>G | missense_variant | c.191G>C | p.Cys64Ser | 0 | 1 | Pathogenic |
| **BRCA1** | 17:41258493 | A>C | missense_variant | c.192T>G | p.Cys64Trp | 0 | 1 | Pathogenic |
| **BRCA1** | 17:41256951 | A>G | missense_variant | c.235T>C | p.Phe79Leu | 1 | 1 | Benign |
| **BRCA1** | 17:41256934 | T>G | missense_variant | c.252A>C | p.Glu84Asp | 0 | 1 | Benign |
| **BRCA1** | 17:41256920 | A>G | missense_variant | c.266T>C | p.Ile89Thr | 0 | 3 | Benign |
| **BRCA1** | 17:41256918 | T>C | missense_variant | c.268A>G | p.Ile90Val | 0 | 2 | Benign |
| **BRCA1** | 17:41223030 | C>T | missense_variant | c.4964G>A | p.Arg1655Lys | 0 | 1 | Benign |
| **BRCA1** | 17:41223023 | C>A | missense_variant | c.4971G>T | p.Lys1657Asn | 1 | 0 | Benign |
| **BRCA1** | 17:41219696 | A>T | missense_variant | c.5066T>A | p.Phe1689Tyr | 2 | 1 | Benign |
| **BRCA1** | 17:41219664 | G>C | missense_variant | c.5098C>G | p.Leu1700Val | 0 | 3 | Benign |
| **BRCA1** | 17:41219652 | C>T | missense_variant | c.5110G>A | p.Glu1704Lys | 0 | 1 | Pathogenic |
| **BRCA1** | 17:41219631 | T>G | missense_variant | c.5131A>C | p.Lys1711Gln | 5 | 8 | Benign |
| **BRCA1** | 17:41219631 | T>C | missense_variant | c.5131A>G | p.Lys1711Glu | 0 | 1 | Benign |
| **BRCA1** | 17:41215954 | A>G | missense_variant | c.5152T>C | p.Cys1718Arg | 0 | 1 | Pathogenic |
| **BRCA1** | 17:41215953 | C>T | missense_variant | c.5153G>A | p.Cys1718Tyr | 0 | 2 | Pathogenic |
| **BRCA1** | 17:41215935 | T>C | missense_variant | c.5171A>G | p.Tyr1724Cys | 0 | 1 | Pathogenic |
| **BRCA1** | 17:41215920 | G>T | missense_variant | c.5186C>A | p.Ala1729Glu | 0 | 1 | Pathogenic |
| **BRCA1** | 17:41215903 | C>A | missense_variant | c.5203G>T | p.Val1735Phe | 0 | 1 | Pathogenic |
| **BRCA1** | 17:41215902 | A>C | missense_variant | c.5204T>G | p.Val1735Gly | 0 | 4 | Pathogenic |
| **BRCA1** | 17:41215896 | T>G | missense_variant | c.5210A>C | p.Tyr1737Ser | 2 | 0 | Benign |
| **BRCA1** | 17:41215389 | C>A | missense_variant | c.5217G>T | p.Trp1739Cys | 0 | 1 | Pathogenic |
| **BRCA1** | 17:41215381 | T>G | missense_variant | c.5225A>C | p.Gln1742Pro | 0 | 1 | Pathogenic |
| **BRCA1** | 17:41209133 | C>T | missense_variant | c.5276G>A | p.Gly1759Glu | 0 | 1 | Pathogenic |
| **BRCA1** | 17:41209110 | G>C | missense_variant | c.5299C>G | p.His1767Asp | 0 | 3 | Pathogenic |
| **BRCA1** | 17:41209092 | C>G | missense_variant | c.5317G>C | p.Ala1773Pro | 0 | 3 | Pathogenic |
| **BRCA1** | 17:41203091 | T>C | missense_variant | c.5384A>G | p.Asn1795Ser | 0 | 1 | Benign |
| **BRCA1** | 17:41201209 | G>C | missense_variant | c.5398C>G | p.Gln1800Glu | 0 | 1 | Benign |
| **BRCA1** | 17:41201197 | T>G | missense_variant | c.5410A>C | p.Met1804Leu | 6 | 14 | Benign |
| **BRCA1** | 17:41201187 | A>G | missense_variant | c.5420T>C | p.Leu1807Pro | 0 | 16 | Pathogenic |
| **BRCA1** | 17:41201184 | C>T | missense_variant | c.5423G>A | p.Cys1808Tyr | 0 | 2 | Pathogenic |
| **BRCA1** | 17:41201179 | C>T | missense_variant | c.5428G>A | p.Ala1810Thr | 0 | 1 | Pathogenic |
| **BRCA1** | 17:41201175 | G>A | missense_variant | c.5432C>T | p.Ser1811Phe | 0 | 1 | Benign |
| **BRCA1** | 17:41201164 | C>T | missense_variant | c.5443G>A | p.Glu1815Lys | 0 | 1 | Benign |
| **BRCA1** | 17:41201146 | G>A | missense_variant | c.5461C>T | p.Leu1821Phe | 1 | 0 | Benign |
| **BRCA1** | 17:41199694 | C>G | missense_variant | c.5496G>C | p.Gln1832His | 0 | 1 | Benign |
| **BRCA1** | 17:41197808 | T>C | missense_variant | c.5542A>G | p.Met1848Val | 0 | 1 | Benign |
| **BRCA1** | 17:41197776 | C>G | missense_variant | c.5574G>C | p.Trp1858Cys | 0 | 3 | Pathogenic |
| **BRCA1** | 17:41197775 | C>T | missense_variant | c.5575G>A | p.Val1859Met | 0 | 2 | Benign |
| **BRCA1** | 17:41197763 | C>T | missense_variant | c.5587G>A | p.Val1863Ile | 0 | 1 | Benign |
| **BRCA1** | 17:41197753 | T>C | missense_variant | c.5597A>G | p.Tyr1866Cys | 0 | 1 | Benign |

BCs: Breast cancer patients; HCs: Healthy controls;

Annotation: Status of the variants in the Findlay et al. study
